# VANet: a medical image fusion model based on attention mechanism to assist disease diagnosis

**DOI:** 10.1186/s12859-022-05072-4

**Published:** 2022-12-19

**Authors:** Kai Guo, Xiongfei Li, Tiehu Fan, Xiaohan Hu

**Affiliations:** 1 College of Instrumentation and Electrical Engineering, Jilin University, Changchun, China; 2 Key Laboratory of Symbolic Computation and Knowledge Engineering of Ministry of Education, Jilin University, Changchun, China; 3 College of Computer Science and Technology, Jilin University, Changchun, China; 4 Department of Radiology, The First Hospital of Jilin University, Changchun, China

**Keywords:** Medical image, Medical image fusion, Attention mechanism, Contextual information, Multi scale feature extraction

## Abstract

****Background**:**

Today’s biomedical imaging technology has been able to present the morphological structure or functional metabolic information of organisms at different scale levels, such as organ, tissue, cell, molecule and gene. However, different imaging modes have different application scope, advantages and disadvantages. In order to improve the role of medical image in disease diagnosis, the fusion of biomedical image information at different imaging modes and scales has become an important research direction in medical image. Traditional medical image fusion methods are all designed to measure the activity level and fusion rules. They are lack of mining the context features of different modes of image, which leads to the obstruction of improving the quality of fused images.

****Method**:**

In this paper, an attention-multiscale network medical image fusion model based on contextual features is proposed. The model selects five backbone modules in the VGG-16 network to build encoders to obtain the contextual features of medical images. It builds the attention mechanism branch to complete the fusion of global contextual features and designs the residual multiscale detail processing branch to complete the fusion of local contextual features. Finally, it completes the cascade reconstruction of features by the decoder to obtain the fused image.

****Results**:**

Ten sets of images related to five diseases are selected from the AANLIB database to validate the VANet model. Structural images are derived from MR images with high resolution and functional images are derived from SPECT and PET images that are good at describing organ blood flow levels and tissue metabolism. Fusion experiments are performed on twelve fusion algorithms including the VANet model. The model selects eight metrics from different aspects to build a fusion quality evaluation system to complete the performance evaluation of the fused images. Friedman’s test and the post-hoc Nemenyi test are introduced to conduct professional statistical tests to demonstrate the superiority of VANet model.

****Conclusions**:**

The VANet model completely captures and fuses the texture details and color information of the source images. From the fusion results, the metabolism and structural information of the model are well expressed and there is no interference of color information on the structure and texture; in terms of the objective evaluation system, the metric value of the VANet model is generally higher than that of other methods.; in terms of efficiency, the time consumption of the model is acceptable; in terms of scalability, the model is not affected by the input order of source images and can be extended to tri-modal fusion.

## Background

As an important auxiliary tool for medical diagnosis, the importance of medical images is self-evident. With the development of sensor technology, the types of medical images are becoming more and more abundant [1, 2]. The information provided to doctors by different types of medical images is usually complementary and how to aggregate these complementary information into one image has become the focus of current research [3–7].

**Fig. 1:**
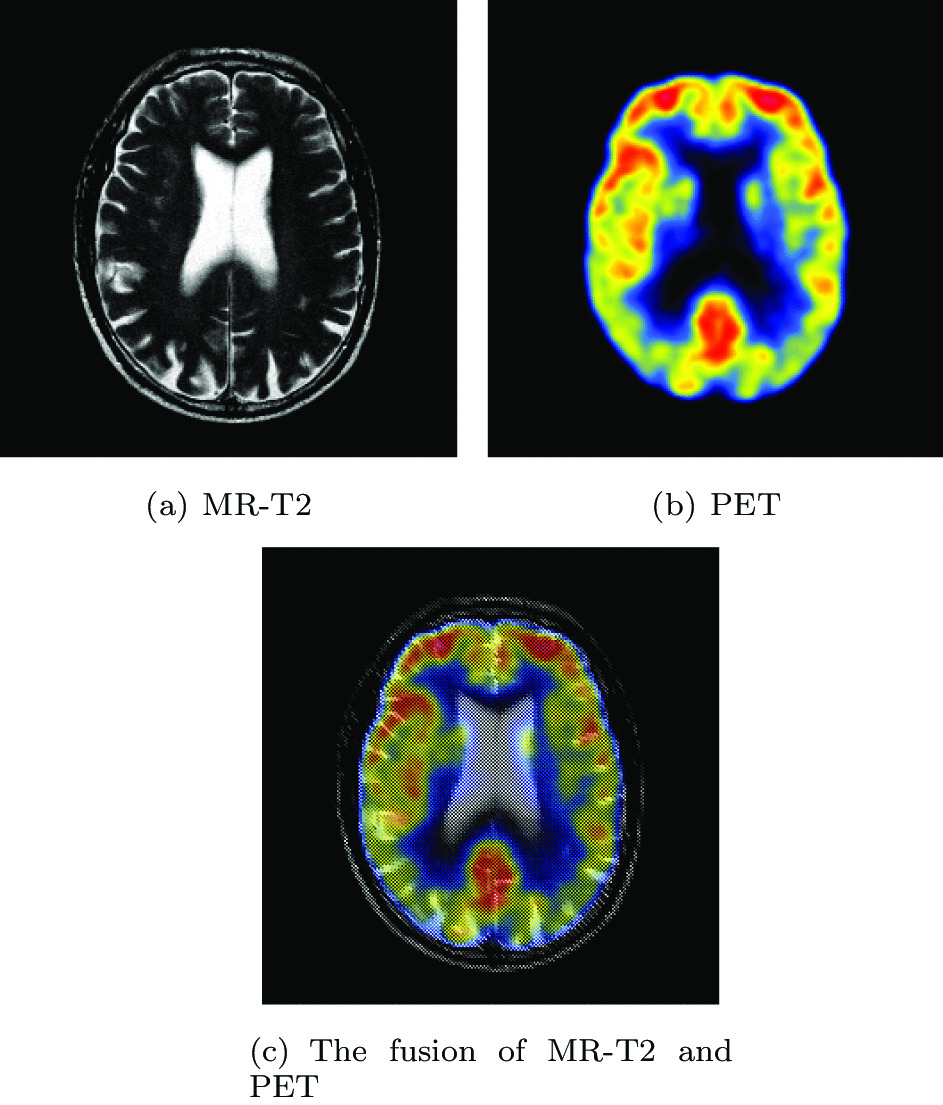
Multi-modal image of a brain metastasis of a bronchial cancer

Figure 1 presents two modal images of a patient with mild Alzheimer’s disease and their fusion results. Figure 1a is the MR-T2 image showing globally widened hemispheric sulci, which is more prominent in parietal lobes. Figure 1b is the PET image that captures signals of markedly abnormal metabolism in brain regions. Weak metabolism occurs in the anterior temporal and posterior parietal regions. The changes tend to be bilateral, but the right hemisphere is more affected than the left, with the posterior cingulate gyrus relatively unaffected. Figure 1c is the fusion result of Fig. 1a and b. Doctors can pay attention to the metabolism of abnormal parts while observing structural changes. It can be seen that medical image fusion is of great significance to clinical diagnosis.

Since the quality of the fused images directly affects the doctor’s judgment of the disease, how to improve the fusion quality of medical images has become an urgent problem to be solved. The quality of fused images depends on the acquisition of image features and the design of fusion rules. Traditional methods usually adopt manual design of feature extraction methods and fusion rules. Although such methods can effectively describe the detailed features of images, they can not acquire the features of images with different modalities. Human-designed image fusion rules focus more on computing weight maps, which integrate pixel activity information from different source images. In traditional fusion methods, the computation of the weight map is achieved by two steps of activity level measurement and weight assignment. Medical images are decomposed by pre-designed filters and their activity is measured by the absolute value of the decomposed coefficients. Then a “choose-max” or “weighted-average” fusion rule is applied to different measurement sources to assign weights.However, this kind of measuring activity and assigning weights are not very stable due to noise, registration and differences in pixel intensities. In order to further improve the performance of the fusion model, scholars have proposed many complex decomposition methods and designed weight allocation strategies carefully. Therefore, these methods are usually designed in steps, breaking the link between activity level measurement and weight assignment.

The medical image fusion method based on deep learning can comprehensively consider the key issues of the fusion image process. This kind of method realizes the direct mapping of source image to weight by encoding the image and completes activity level measurement and weight assignment in an ”optimal” way via learning network parameters, which enhances the correlation between activity level measurement and weight assignment effectively. In all deep learning algorithms, improved algorithms based on autoencoders (AE) [8–10], generative adversarial networks (GAN) [11, 12] and convolutional neural networks (CNN) [13–15] are popular in medical image fusion. Song et al. proposed MSDNet and applied it to the extraction of medical image features [16]. The multiplexing of features enhanced the expression of important information in the fused image; Kang et al. regarded the fusion of PET and MR images as a min-max optimization problem with respect to the generator and the discriminator [17]. They proposed TAcGAN model to enhance the structural features of fused images through a game of generator and discriminator, while preserving part of the information of SPECT images. Zhang et al. proposed a general fusion framework based on convolutional neural network called IFCNN [18]. IFCNN can obtain the salient features of medical images without being limited by the number of source images. The fused images preserves important features from different images better.

Although the above methods improve the fusion quality of medical images, their improvement is limited. This is because they only focus on image fusion itself, ignoring the significance of medical image fusion. Medical image fusion focuses on the global and local effects of abnormal tissue on medical images, which are often reflected in the contextual information of images. Therefore, how to obtain image context information has become the top priority of current research. In order to address this issue, we propose a new medical image fusion model on deep learning, called VAnet. The VAnet model has two most important parts, the encoder and the fusion network. The encoder consists of five convolutional pooling blocks of the VGG-16 network, which can sufficiently capture the contextual information of medical images. The fusion network adopts the method of combining residual multi-scale feature extraction and attention mechanism to realize the enhancement of salient features and the preservation of texture detail information.

## Methods

### VAnet

#### Overview

**Fig. 2:**
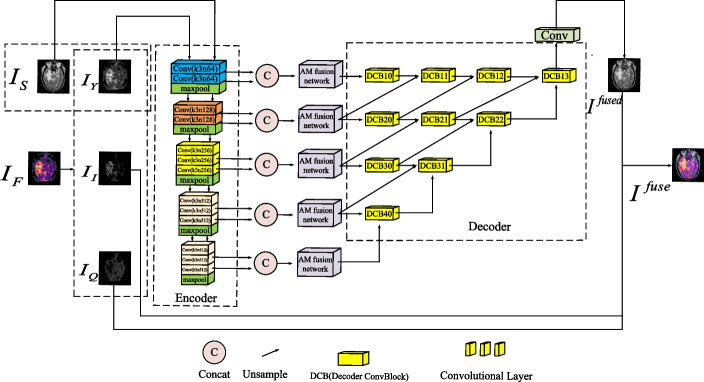
Schematic diagram of the VAnet model

VAnet is a new type of medical image fusion model. It consists of three parts: encoder, AM fusion network and decoder. In Fig. 2, the encoder consists of five coding blocks, which are corresponding to five blocks of VGG-16, respectively. The five feature maps obtained from five blocks contain all the contextual semantic information of the image. Then the feature maps are put into the AM fusion network for multi-scale deep feature fusion. The AM fusion network consists of the attention mechanism branch and the residual multi-scale detail fusion branch. The attention mechanism branch consists of the channel attention mechanism block and five convolution blocks. Among them, the channel attention mechanism block can suppress noise, especially functional images. The residual multi-scale detail fusion branch includes three convolution blocks and a multi-scale detail fusion block. Among them, the multi-scale detail fusion block can completely compensate for the loss of detail caused by the pooling operation in the attention mechanism. Finally, the fused feature map will be input to the decoder to reconstruct the fused image.

#### Encoder

Traditional encoders tend to ignore the context information of feature maps in feature extraction. Facts have proved that the pathological characteristics of tissues are not only reflected in a certain independent part, but also in its contextual information. Therefore, we select the VGG-16 network that can obtain context information in the encoder.

**Fig. 3:**
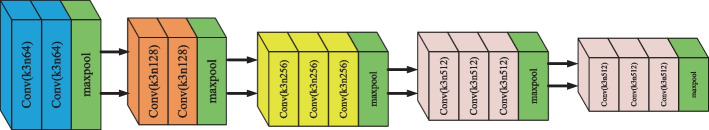
The structure of the encoder of the VAnet model

As shown in Fig. 3, VGG-16 contains five blocks. Its biggest feature is that it can obtain information about the image context. The first two blocks consist of two convolutional layers and one max-pooling layer, respectively. The last three blocks consist of three convolutional layers and one max-pooling layer, respectively. The stacking of the two can easily form a deeper network structure to obtain more complete and deeper contextual information. The kernel size of all convolutional layers is 3 × 3 and the size of max pooling layers is 2 × 2. The first four blocks have different numbers of output channels; the fourth and last blocks have the same number of output channels.

#### AM fusion network

**Fig. 4:**
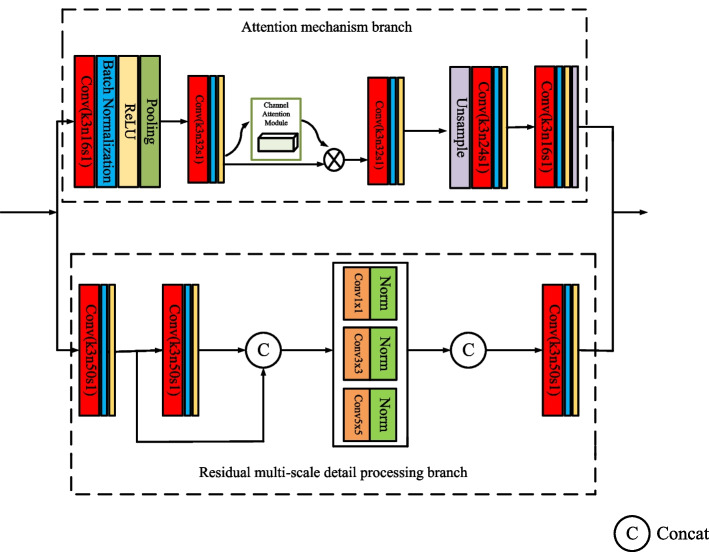
The structure of the AM fusion network

AM fusion network is the core part of the VAnet model. The extraction of important features and their associated features, the suppression of noise and the preservation of texture details all rely on the fusion network. In Fig. 4, AM fusion network consists of the attention mechanism branch and the residual multi-scale detail processing branch.

**Fig. 5:**
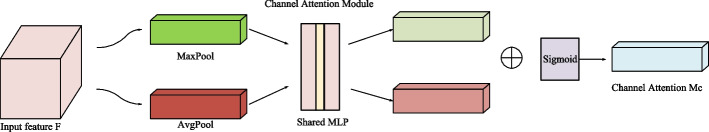
The structure of the channel attention block

*Attention mechanism branch* Attention mechanism branch is composed of five convolutional blocks and a channel attention mechanism block. Each convolutional block is composed of a convolutional layer, a batch normalized layer and a ReLU activation function. The kernel of the convolution layer in all convolution blocks is 3 × 3. In the first convolution block, a pooling layer is added after the activation function to reduce the feature dimension. In the fourth convolution block, we add an unsampled layer before the convolution layer to restore the feature dimension. A channel attention mechanism block is added behind the second convolution block and its working principle is shown in Fig. 5.

In Fig. 5, the size of the input feature map F is $H \times W \times C$, which is put into the max pooling layer and the average pooling layer to obtain two $1 \times 1 \times C$ feature maps. Then the two feature maps are fed into a two-layer shared neural network for feature extraction. The number of neurons in the first layer of the network is *C*/*r* and the ReLU function is selected as the activation function. The number of neurons in the second layer of the network is C. The element-wise operation is performed on the features obtained by the shared neural network and the final channel attention feature Mc is generated after the sigmoid activation operation.

*Residual multi-scale detail processing branch* After the image is branched by the attention mechanism, the detailed information will be lost, which will affect the fusion result of the image. In order to avoid the above situation, the residual multi-scale detail fusion block is designed. The residual multi-scale detail processing block includes a set of residual convolution blocks, a multi-scale detail fusion block and a convolution block. Among them, the residual convolution block is designed to prevent gradient explosion. The convolution kernels of all convolution blocks are set to 3 × 3. In the multi-scale detail fusion block, we use three different convolution kernels. Different convolution kernels can fuse detailed information of different scales. The selection of the convolution kernel is shown in Fig. 4. Among them, a 1 × 1 convolution kernel filter is used to process the information of different channels at the same location. Filters with 3 × 3 and 5 × 5 convolution kernels are used to process the information of the surrounding channels at the same location. The reason why a filter with a larger convolution kernel is not used to process the surrounding information at the same position is due to the consideration of the computational complexity of the model. A large convolution kernel will bring more computation to the model and affect the computational performance of the model seriously.

#### Decoder

The decoder is based on a nested connection architecture. Inspired by UNet++, we simplified its structure. As shown in Fig. 2, the decoder consists of ten convolutional blocks. Each convolution block is composed of two convolution layers with convolution kernel of 3 × 3. The cross-layer link connects the multi-scale depth features in the decoder. The output of the decoder is a reconstructed image fused with multi-scale features.

#### Loss function

In order to improve the fusion effect of the VAnet model, we use the structural similarity (SSIM) loss function, the mean squared variance (MSE) loss function and the total variation (TV) loss function to form a mixed loss function. The description of the hybrid loss function is as follows

1 $$\begin{aligned} {L_{total}} = \alpha {L_{SSIM}} + \beta {L_{MSE}} + {L_{TV}} \end{aligned}$$

where $\alpha$ and $\beta$ are the balance parameters. The SSIM loss function is used to measure the loss of texture details of the source image during the fusion process. The MSE loss function is used to predict the pixel-to-pixel loss between the fused image and source images. The introduction of TV loss function aims to maintain the smoothness of the image and suppress noise. The structural similarity loss function is described as

2 $$\begin{aligned} {L_{SSIM}} = \sum \limits _{i = 1}^N {\left( {1 - SSIM\left( {{I^{fused}},{I^{source}}} \right) } \right) } \end{aligned}$$

where ${I^{fused}}$ represents the fused image and ${I^{source}}$ represents the source images. *N* is the size of the batch. $SSIM( \cdot )$ is used to calculate the structural similarity between images. The closer the SSIM value is to 1, the more detailed information of the source image is contained in the fused image. The MSE loss function is defined as follows

3 $$\begin{aligned} {L_{MSE}} = \frac{1}{{WH}}\sum \limits _{x = 1}^W {\sum \limits _{y = 1}^H {{{\left( {I_{x,y}^{source} - I_{x,y}^{fused}} \right) }^2}} } \end{aligned}$$

where *W* and *H* are width and height of the image, respectively. (*x,y*) is the pixel position of the image. The total vision loss function is described as

4 $$\begin{aligned} {L_{TV}} = \sum \limits _{i,j} {\left( {{{\left( {I_{x,j - 1}^{fused} - I_{x,j}^{fused}} \right) }^2} + {{\left( {I_{x + 1,j}^{fused} - I_{x,j}^{fused}} \right) }^2}} \right) } \end{aligned}$$

### Dataset and Experimental environment

The experimental data in the article are selected from the AANLIB database. 100 pairs of cross-modally registered brain abnormalities medical images are downloaded and cropped into 11960 patch pairs as the training set for the VANet model. The size of each patch is set to 84x84. This operation not only ensures the diversity of training data, but also enhances the robustness of VAnet. As for the test data, we randomly selected two sets of images from each of the 4 diseases to complete the test on VAnet. The training and testing of the VAnet model are all tested on a machine equipped with a 2.4 GHz Intel Core i7-11800H CPU (32G RAM) and a GeForce RTX 3070 GPU.

### Comparison algorithm and metrics

In this section, eleven medical image fusion methods are selected for comparison with VAnet. These eleven algorithms are GFF [19], NSCT [20], IGM [21], LPSR [22], WLS [23], CSR [24], LRD [25], TLAYER [26], CSMCA [27], LATLRR [28] and DTNP [29]. Among them, GFF, NSCT, IGM, LRD and TLAYER are traditional image fusion methods. WLS and CSMCA are deep learning fusion methods. LPSR is a fusion method based on sparse representation classes. CSR is a fusion method combining neural network and sparse representation. LATLRR is based on a low-rank decomposition fusion method. DTNP is a fusion method that combines dynamic threshold and wavelet transform. The source codes of all comparison algorithms come from the Internet and the settings of each algorithm parameters are recommended by the corresponding authors.

In order to evaluate the performance of VAnet, we selected eight evaluation metrics to analyze the fused images of all algorithms. The eight metrics are Qw [30], Qe, SSIM [31], VIF [32], FMI [33], LABF [34], NABF [35] and NCIE [36]. Among them, Qw and Qe are derived from the Piella model. SSIM is used to measure the structural similarity between the fused image and the source image. VIF stands for visual evaluation of fused images. LABF, NABF, FMI and NCIE are representative metrics for evaluating image fusion in information theory.

### Training details

The training of the VANet model involves many parameters, including batch_size, learning rate, epoch, and the balance parameter in the loss function. The settings of these parameters can have a profound effect on the fusion effect. Therefore, the analysis of these parameters has important research significance.

#### Batch_size

batch_size refers to the number of samples selected for a training and its size affects the optimization degree and speed of the model. Since the data for training VAnet model is relatively large, putting all the data into the network at one time will definitely cause a memory explosion. Therefore, batch_size needs to be introduced to solve this problem. However, the value of batch_size can not be too small. If it is too small, the learning will be random and the model will not converge. Considering the hardware environment and memory capacity of the experiment, according to Leslie’s theory, we set the value of batch_size to 64.

#### Epoch

**Fig. 6:**
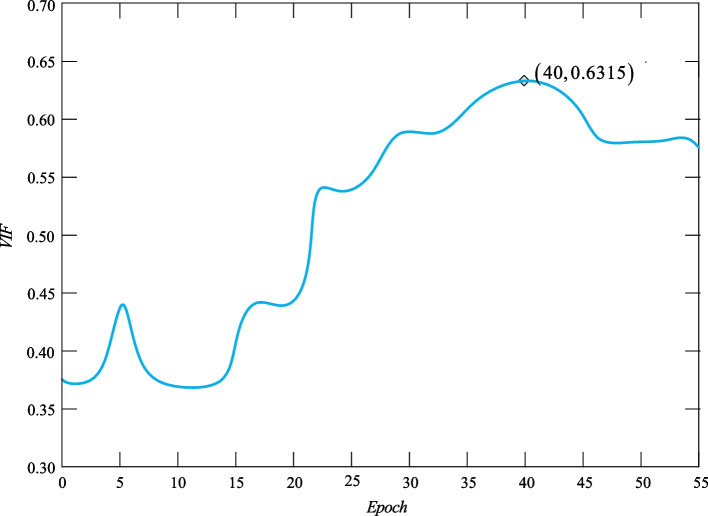
The changing trend of epoch

Epoch is an important parameter that controls the number of weight update iterations and the weight update iteration directly affects the fit and convergence of the model. In the training of the VANet model, it is not enough to train all the data in one iteration to get the model into the best fit state. Therefore, it is necessary to set an appropriate epoch value to improve the stability of the model and the effect of image fusion. VIF is a metric that evaluates image quality from the perspective of information communication and sharing based on the statistical properties of natural scenes. Since the evaluation accuracy of this metric is related to the image itself and the distortion channel of the human visual system, it is very appropriate to choose it to assist in completing the determination of the value of epoch. Figure 6 shows the trend of VIF with the transformation of the epoch.

In Fig. 6, we give the average value of VIF for 50 pairs of medical fused images. When epoch is set to 40, the corresponding images average VIF value reaches the maximum and the fused image obtained is more in line with human visual perception. Therefore, we set the value of epoch to 40 to complete the training of the VANet model.

#### learning rate

The learning rate is an important parameter of the VANet model, which affects the convergence of the model. If the learning rate is too large, the model will oscillate and not converge. If the learning rate is too small, the model will converge slowly. Based on the actual situation, we chose the exponential decay learning rate. The formula is as follows

5 $$\begin{aligned} lr = l{r_{base}} * l{r_{decay}}^{epoch} \end{aligned}$$

where $l{r_{base}}$ is the initial value of the learning rate and $l{r_{decay}}$ is the decay rate of learning rate. According to prior knowledge, the initial value of the learning rate is set to 0.1, and the decay value of the learning rate is set to 0.99.

#### Hyperparameters

**Fig. 7:**
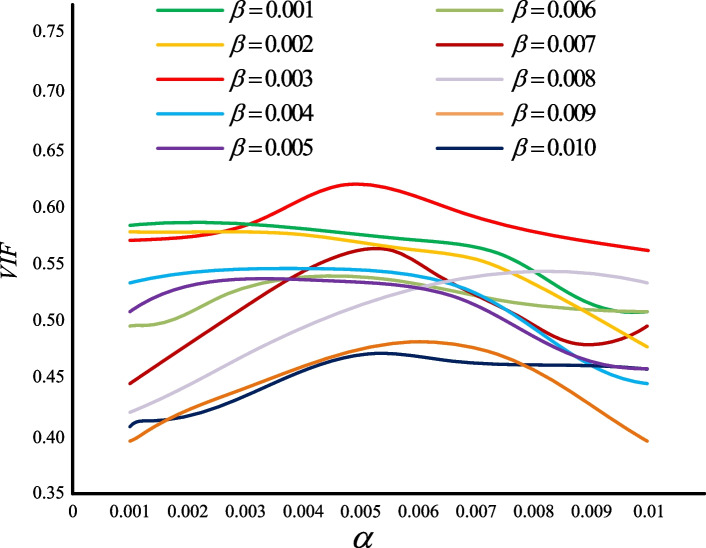
The Hyperparameters change trend graph

In the loss function of the VANet model, there are two hyperparameters $\alpha$ and $\beta$, which are used to adjust SSIM loss function and MSE loss function respectively. With reference to other scholars setting hyperparameters for deep learning, the values of $\alpha$ and $\beta$ are set between 0 and 0.01. Given the role of the two loss functions in the training process, we chose the evaluation metric VIF that related to the human eye perception to assist in determining the values of the hyperparameters $\alpha$ and $\beta$. Figure 7 shows the trend of VIF with $\alpha$ and $\beta$.

In Fig. 7, we give the average value of VIF for 50 pairs of medical fused images. Obviously, when $\alpha$ is set to 0.005 and $\beta$ is set to 0.003, the average VIF value of the corresponding image reaches the maximum value, which best meets the requirements of VANet model training.

## Results

The test data are derived from the following five diseases, which are subacute stroke, hypertensive encephalopathy, cavernous hemangioma, metastatic bronchogenic carcinoma and mild Alzheimer’s disease. Two pairs of the source images are selected for each disease to prove the effectiveness and superiority of our fusion model.

### Subacute stroke: loss of sensation

**Fig. 8:**
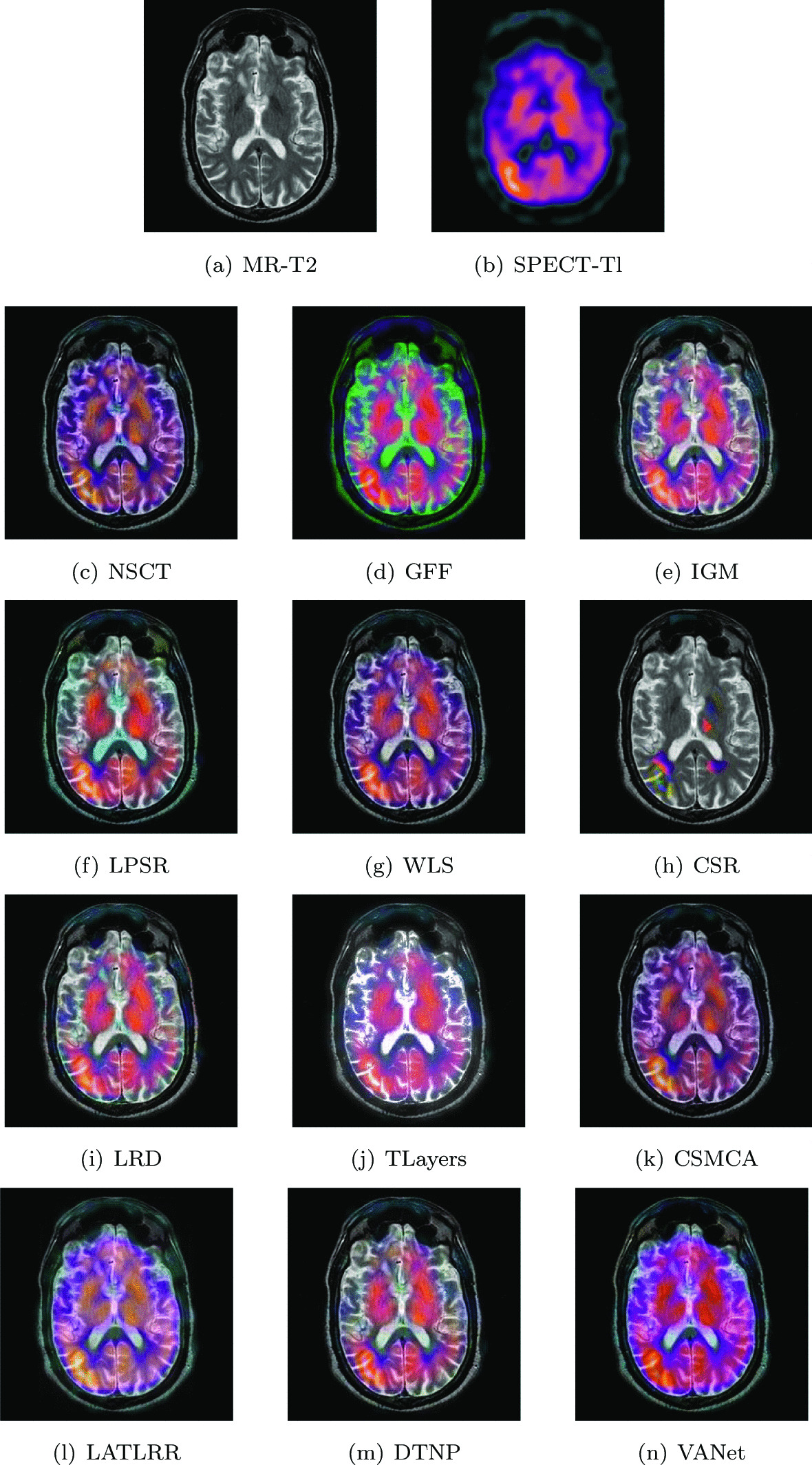
The first set of fused MRI-SPECT images from 9 methods on subacute stroke

**Fig. 9:**
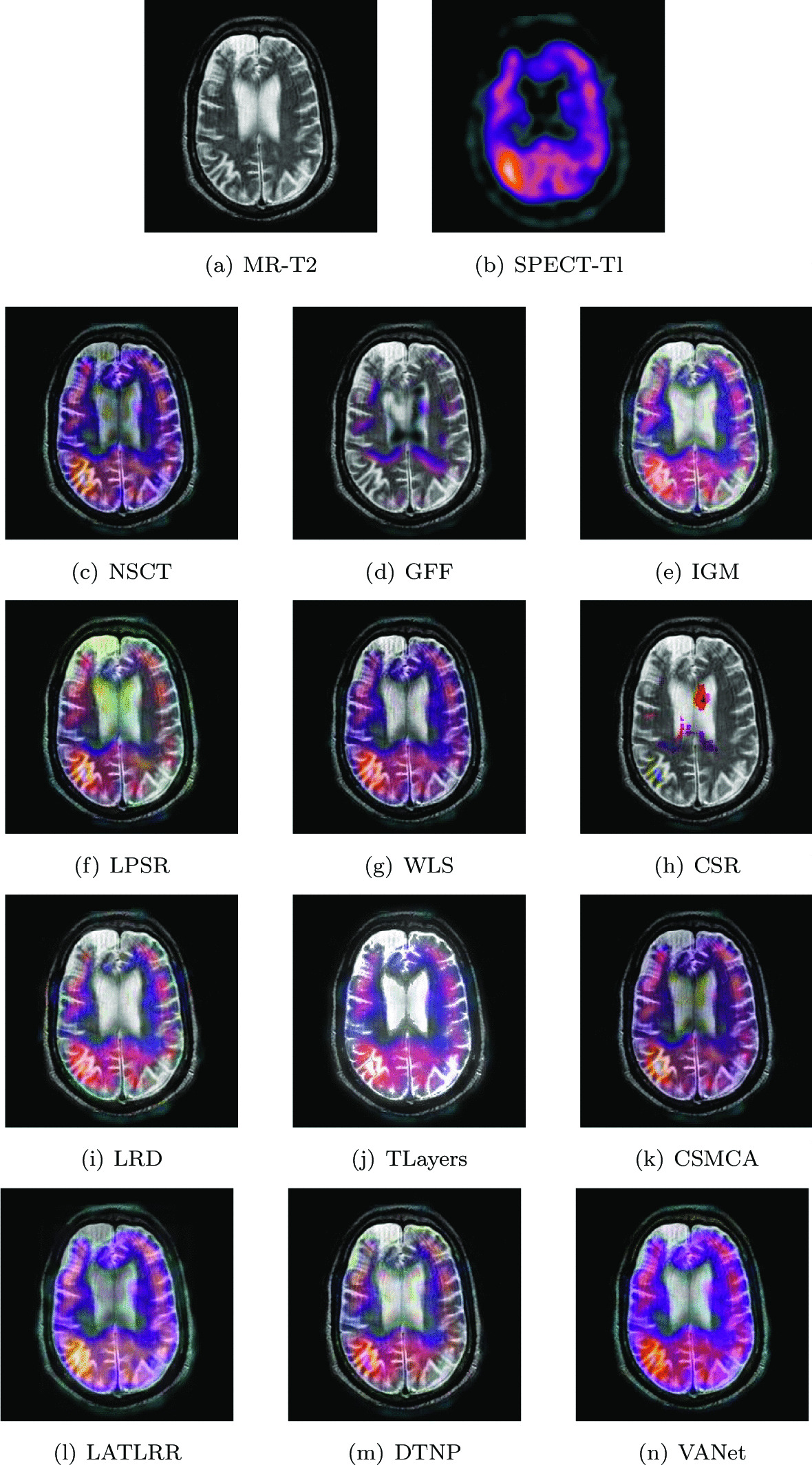
The second set of fused MRI-SPECT images from 9 methods on subacute stroke

The two sets of source images in this section are from a 65-year-old patient with subacute stroke. He is right-handed with mild left hemiplegia and atrial fibrillation. When he felt a tingling pain in his left arm, he went to the hospital and found that he could not explore the left half of the space. In his two sets of MR images, the cerebrospinal fluid left behind by the liquefaction and necrosis of the old infarct showed hyperintensity and successfully replaced the frontal pole. Hyperperfusion appears on the corresponding SPECT images. Figures 8 and 9 show the fusion results of all algorithms on two sets of subacute stroke images. The fused image based on CSR model almost loses the ability to describe functional information. The fused images obtained by LRD, IGM, TLayers and DTNP algorithms can not completely describe the blood flow level. The fused images obtained by GFF and LPSR algorithms have serious distortion. The brightness of the fused images obtained by NSCT, WLS and CSMCA is dark, which is not conducive to the description of the structural information of the image. The fused image obtained by LATLRR algorithm has serious blurring. The fused image obtained by VANet model can clearly describe the blood flow situation of the tissue, while retaining the key information in the MR images.

Tables 1 and 2 give the objective performance of different algorithms on the fusion of the above two sets of medical images, respectively. The VANet model achieves optimal values on all objective evaluation metrics. From both subjective and objective perspectives, the subacute stroke images fused by VANet model can provide doctors with complete information about the diseased tissue and help doctors complete the diagnosis as soon as possible.

**Table 1:** The objective evaluation scores about group 1 fused images

Methods	Metrics								
	*Q*_*w*_	*Q*_*e*_	SSIM	VIF	FMI	NCIE	LABF	NABF	Time
NSCT	0.7751 (10.59%)	0.8586 (4.66%)	0.7595 (10.23%)	0.5421 (14.96%)	0.6057 (11.95%)	0.8060 (2.58%)	0.1003 (− 14.96%)	0.0271 (− 31.37%)	0.4843 (5th)
GFF	0.6792 (26.20%)	0.6961 (29.09%)	0.6570 (27.42%)	0.5194 (19.98%)	0.5544 (22.31%)	0.8064 (2.53%)	0.1295 (− 34.13%)	0.0189 (− 1.59%)	0.0527 (2nd)
IGM	0.7639 (12.21%)	0.8143 (10.35%)	0.7684 (8.95%)	0.5956 (4.63%)	0.5882 (15.28%)	0.8072 (2.43%)	0.1247 (− 31.60%)	0.0257 (− 27.63%)	1.9045 (7th)
LPSR	0.7974 (7.50%)	0.8708 (3.19%)	0.8017 (4.43%)	0.5920 (5.27%)	0.6393 (6.07%)	0.8071 (2.44%)	0.0945 (− 9.74%)	0.0294 (− 36.73%)	0.1302 (3rd)
WLS	0.7768 (10.35%)	0.8590 (4.61%)	0.7392 (13.25%)	0.5417 (15.05%)	0.6361 (6.60%)	0.8066 (2.50%)	0.0957 (− 10.87%)	0.0294 (− 36.73%)	0.0276 (1st)
CSR	0.7918 (8.26%)	0.8869 (1.32%)	0.7889 (6.12%)	0.5264 (18.39%)	0.6713 (1.01%)	0.8126 (1.75%)	0.1103 0.0861	0.0323 (− 42.41%)	16.1686 (10th)
LRD	0.7707 (13.22%)	0.8484 (5.92%)	0.7837 (6.83%)	0.6077 (2.55%)	0.6298 (7.67%)	0.8079 (2.34%)	(− 0.93%) (− 22.67%)	0.0265 (− 29.81%)	33.4586 (11th)
TLayers	0.6816 (25.76%)	0.7167 (25.38%)	0.7162 (16.89%)	0.5125 (21.60%)	0.5107 (32.78%)	0.8071 (2.44%)	0.1075 (− 20.65%)	0.0315 (− 40.95%)	0.6064 (6th)
CSMCA	0.7842 (9.31%)	0.8656 (3.81%)	0.7233 (15.75%)	0.5249 (18.73%)	0.6193 (9.49%)	0.8066 (2.50%)	0.1042 (− 18.14%)	0.0213 (− 12.68%)	35.8234 (12th)
LATLRR	0.6895 (24.32%)	0.6966 (29.00%)	0.7334 (14.15%)	0.5819 (7.10%)	0.5444 (24.56%)	0.8065 (2.52%)	0.1220 (− 30.08%)	0.0199 (− 6.53%)	8.2307 (8th)
DTNP	0.7982 (7.39%)	0.8709 (3.18%)	0.7990 (4.78%)	0.5925 (5.18%)	0.6357 (6.67%)	0.8075 (2.39%)	0.0894 (− 4.59%)	0.0298 (− 37.58%)	14.0949 (9th)
VANet	0.8572 (1st)	0.8986 (1st)	0.8372 (1st)	0.6232 (1st)	0.6781 (1st)	0.8268 (1st)	0.0853 (1st)	0.0186 (1st)	0.1568 (4th)

**Table 2:** The objective evaluation scores about group 2 fused images

Methods	Metrics								
	*Q*_*w*_	*Q*_*e*_	SSIM	VIF	FMI	NCIE	LABF	NABF	Time
NSCT	0.7348 (10.59%)	0.8307 (7.04%)	0.7361 (12.53%)	0.5699 (11.93%)	0.6032 (9.43%)	0.8056 (2.23%)	0.1168 (− 17.47%)	0.0286 (− 30.07%)	0.4715 (5th)
GFF	0.7522 (8.03%)	0.8571 (3.75%)	0.7892 (4.95%)	0.5621 (13.49%)	0.6152 (7.30%)	0.8114 (1.50%)	0.1005 (− 4.08%)	0.0323 (− 38.08%)	0.0552 (2nd)
IGM	0.7554 (7.52%)	0.7950 (11.85%)	0.7938 (4.35%)	0.6258 (1.93%)	0.5850 (12.84%)	0.8072 (2.03%)	0.1470 (− 34.42%)	0.0238 (− 15.97%)	1.8972 (8th)
LPSR	0.7989 (1.71%)	0.8696 (2.25%)	0.8098 (2.28%)	0.6243 (2.18%)	0.6354 (3.89%)	0.8070 (2.06%)	0.0990 (− 2.62%)	0.0303 (− 33.99%)	0.0205 (1st)
WLS	0.7768 (4.61%)	0.8464 (5.06%)	0.5782 (43.25%)	0.5724 (11.44%)	0.6226 (6.02%)	0.8065 (2.12%)	0.0991 (− 2.72%)	0.0367 (− 45.50%)	0.1343 (3rd)
CSR	0.7812 (4.02%)	0.8719 (1.98%)	0.8148 (1.66%)	0.5814 (9.72%)	0.6474 (1.96%)	0.8120 (1.43%)	0.0988 (− 2.43%)	0.0358 (− 44.13%)	13.8835 (9th)
LRD	0.7769 (4.60%)	0.8323 (6.84%)	0.7786 (6.38%)	0.6267 (1.79%)	0.6324 (4.38%)	0.8083 (1.89%)	0.1159 (− 16.82%)	0.0256 (− 21.88%)	33.2710 (11th)
TLayers	0.6931 (17.24%)	0.7146 (24.43%)	0.7631 (8.54%)	0.5539 (15.17%)	0.5142 (28.37%)	0.8070 (2.06%)	0.1163 (− 17.11%)	0.0341 (− 41.35%)	0.5785 (6th)
CSMCA	0.7521 (8.04%)	0.8403 (5.82%)	0.7101 (16.65%)	0.5632 (13.26%)	0.6081 (8.55%)	0.8059 (2.20%)	0.1237 (− 22.07%)	0.0213 (− 6.10%)	51.6378 (12th)
LATLRR	0.7630 (6.50%)	0.6861 (29.60%)	0.7602 (8.96%)	0.6119 (4.25%)	0.5478 (20.50%)	0.8061 (2.17%)	0.1873 (− 48.53%)	0.0203 (− 1.48%)	8.0366 (7th)
DTNP	0.7892 (2.97%)	0.8601 (3.38%)	0.8073 (2.60%)	0.6207 (2.77%)	0.6314 (4.55%)	0.8077 (1.97%)	0.0969 (− 0.52%)	0.0295 (− 32.20%)	14.4718 (10th)
VANet	0.8126 (1st)	0.8892 (1st)	0.8283 (1st)	0.6379 (1st)	0.6601 (1st)	0.8236 (1st)	0.0964 (1st)	0.0200 (1st)	0.1622 (4th)

#### Hypertensive encephalopathy

**Fig. 10:**
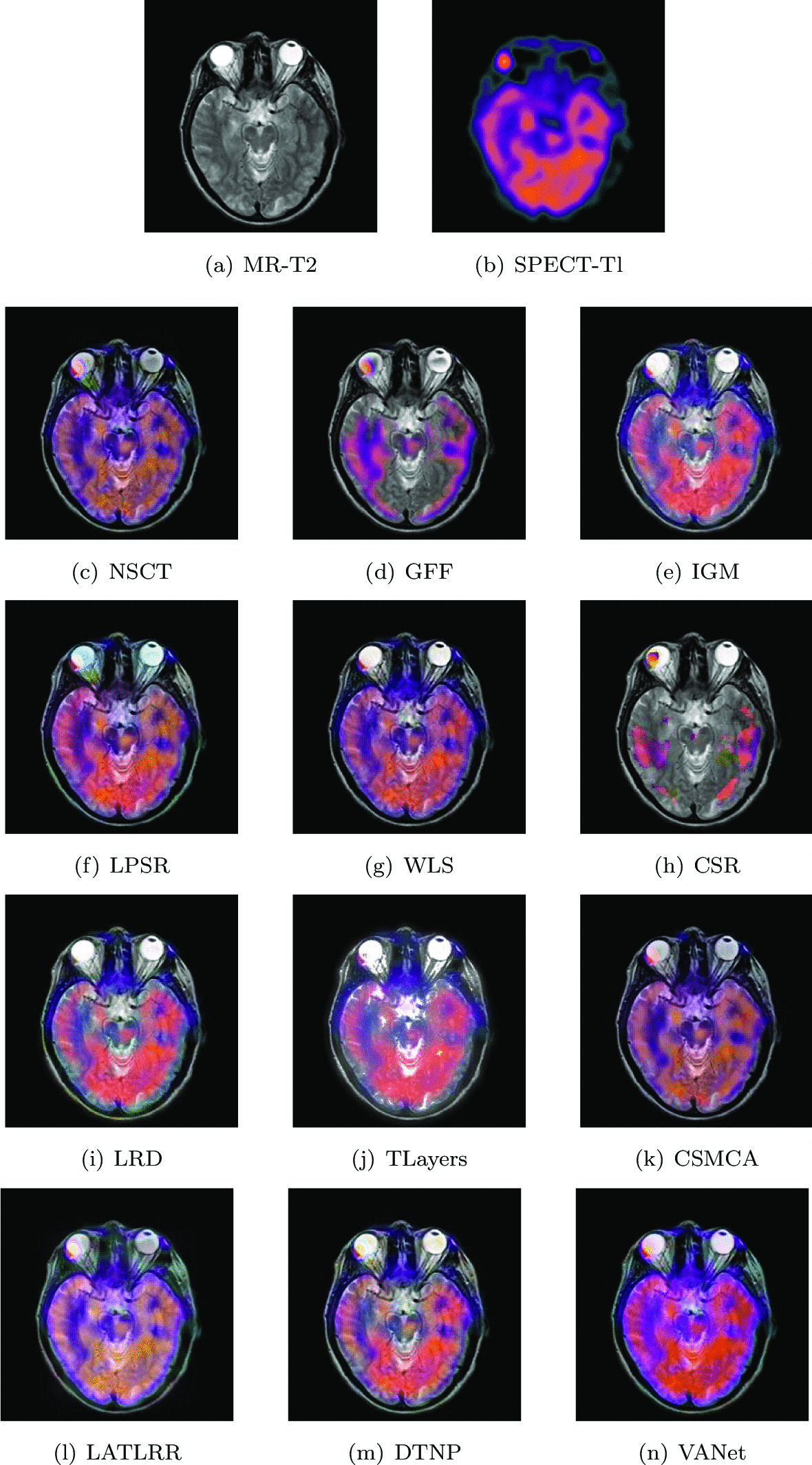
The first set of fused MRI-SPECT images from 9 methods on hypertensive encephalopathy

**Fig. 11:**
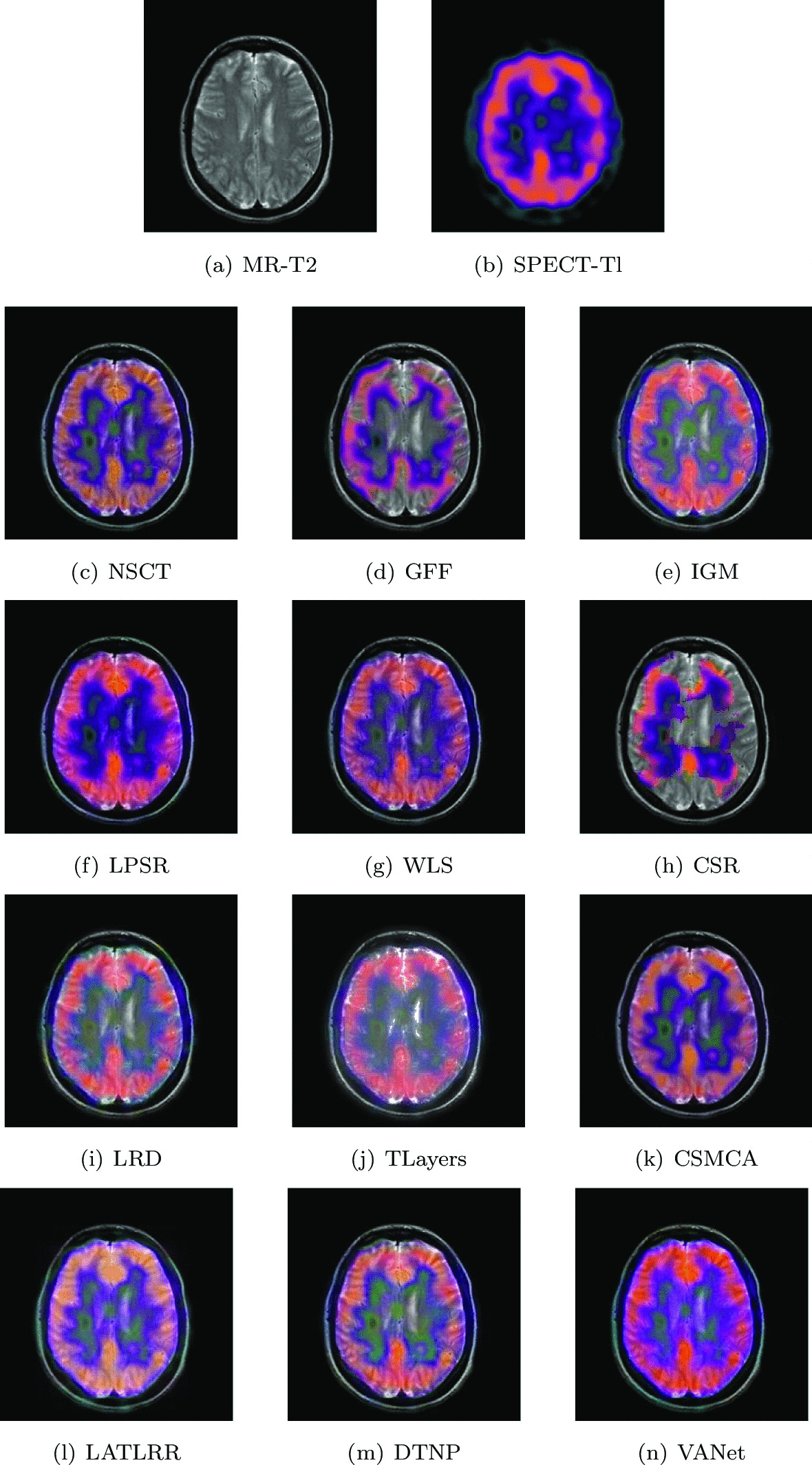
The second set of fused MRI-SPECT images from 9 methods on hypertensive encephalopathy

Two sets of source images in Figs. 10 and 11 are from a young woman that has acute arterial hypertension. In her MR− T2 images, bilateral temporal and occipital lesions can be clearly seen. Early perfusion abnormalities are obvious at higher levels in her SPECT-Tl image. In order to observe the lesion tissue and its perfusion better, the two sets of images are selected for fusion on 12 algorithms and the fusion results are shown in Figs. 10 and 11, respectively. The fused images obtained by NSCT, WLS and CSMCA algorithms have a dim brightness and lose the energy information in the SPECT image. The fused images obtained by GFF, LPSR and CSR algorithms have serious distortion. The fused image obtained by TLayers algorithm is very blurry and can not describe the texture information. The fused images obtained based on IGM, LRD and DTNP algorithms have a large brightness, which affects the expression of some detailed information. The fused image obtained by LATLRR algorithm loses part of the color information, which affects the description of the blood flow information. The fused image obtained by VANet model can characterize the diseased tissue and its blood flow better.

In Tables 3 and 4, it can be seen that the VAnet model is outstanding on Qw, Qe, SSIM, LABF, NABF and NCIE. On VIF and FMI, the performance of VAnet is lower than that of the LATLRR algorithm and the CSR model respectively, which may be related to the feature extraction method. However, the fused images obtained by LATLRR algorithm and CSR model lack different color information, which makes them unable to provide reliable information for doctors. In contrast, the images fused by VANet model can obtain more complete color information,which may be helpful for treating hypertensive encephalopathy.

**Table 3:** The objective evaluation scores about group 3 fused images

Methods	Metrics								
	*Q*_*w*_	*Q*_*e*_	SSIM	VIF	FMI	NCIE	LABF	NABF	Time
NSCT	0.7433 (7.04%)	0.8220 (4.28%)	0.6955 (28.68%)	0.5658 (10.32%)	0.6304 (12.67%)	0.8057 (1.79%)	0.1205 (− 15.93%)	0.0290 (− 30.34%)	0.4928 (5th)
GFF	0.7402 (7.48%)	0.8363 (2.50%)	0.7037 (24.21%)	0.5240 (19.12%)	0.7010 (1.33%)	0.8094 (1.32%)	0.1146 (− 11.61%)	0.0283 (− 28.62%)	0.0548
									(2nd)
IGM	0.7484 (6.31%)	0.8026 (6.80%)	0.7032 (24.30%)	0.5891 (5.96%)	0.6508 (9.14%)	0.8068 (1.65%)	0.1380 (− 26.59%)	0.0255 (− 20.78%)	2.1848 (7th)
LPSR	0.7867 (1.13%)	0.8454 (1.40%)	0.7645 (14.33%)	0.6133 (1.78%)	0.6863 (3.50%)	0.8046 (1.70%)	0.1118 (− 9.39%)	0.0299 (− 32.44%)	0.0245 (1st)
WLS	0.7675 (3.66%)	0.8297 (3.31%)	0.7092 (23.25%)	0.5568 (12.10%)	0.6854 (3.63%)	0.8064 (1.70%)	0.1113 (− 8.98%)	0.0315 (− 35.87%)	0.1407 (3rd)
CSR	0.7514 (5.88%)	0.8389 (2.18%)	0.7202 (21.36%)	0.5168 (20.78%)	0.7026 (1.10%)	0.8101 (1.23%)	0.1078 (− 6.03%)	0.0370 (− 45.41%)	15.2815 (10th)
LRD	0.7729 (2.94%)	0.8186 (4.72%)	0.7245 (20.65%)	0.5981 (4.36%)	0.6682 (6.30%)	0.8073 (1.59%)	0.1290 (− 21.47%)	0.0232 (− 12.93%)	33.4755 (11th)
TLayers	0.6445 (23.44%)	0.6710 (27.75%)	0.5779 (51.25%)	0.5068 (23.16%)	0.5816 (22.13%)	0.8069 (1.64%)	0.1766 (− 42.63%)	0.0376 (− 46.28%)	0.5856 (6th)
CSMCA	0.7644 (4.08%)	0.8357 (2.57%)	0.8616 (1.45%)	0.5616 (11.15%)	0.6757 (5.12%)	0.8062 (1.72%)	0.1290 (− 21.47%)	0.0206 (− 1.94%)	36.0302 (12th)
LATLRR	0.6702 (18.71%)	0.6808 (25.91%)	0.7026 (24.41%)	0.6243 (− 0.02%)	0.5949 (19.40%)	0.8062 (1.72%)	0.1789 (− 43.38%)	0.0217 (− 6.91%)	8.1392 (8th)
DTNP	0.7813 (1.83%)	0.8443 (1.53%)	0.7690 (13.67%)	0.6112 (2.13%)	0.6627 (7.18%)	0.8068 (1.65%)	0.1017 (− 0.39%)	0.0317 (− 36.27%)	13.9342 (9th)
VANet	0.7956 (1st)	0.8572 (1st)	0.8741 (1st)	0.6242 (2nd)	0.7103 (1st)	0.8201 (1st)	0.1013 (1st)	0.0202 (1st)	0.1573 (4th)

**Table 4:** The objective evaluation scores about group 4 fused images

Methods	Metrics								
	*Q*_*w*_	*Q*_*e*_	SSIM	VIF	FMI	NCIE	LABF	NABF	Time
NSCT	0.7958 (2.17%)	0.8520 (0.77%)	0.8083 (6.05%)	0.6728 (11.56%)	0.6509 (5.93%)	0.8060 (1.38%)	0.1228 (− 0.57%)	0.0289 (− 47.06%)	0.5051 (5th)
GFF	0.7622 (6.68%)	0.8407 (2.13%)	0.7531 (13.82%)	0.6150 (22.05%)	0.6111 (12.83%)	0.8071 (1.24%)	0.1513 (− 19.30%)	0.0263 (− 41.83%)	0.0560 (2nd)
IGM	0.7165 (13.48%)	0.7414 (15.80%)	0.6855 (25.05%)	0.6234 (20.40%)	0.6550 (5.27%)	0.8067 (1.29%)	0.2033 (− 39.94%)	0.0222 (− 30.08%)	2.1543 (7th)
LPSR	0.7806 (4.16%)	0.8350 (2.83%)	0.8451 (1.43%)	0.7085 (5.94%)	0.6728 (2.48%)	0.8060 (1.38%)	0.1399 (− 12.72%)	0.0327 (− 53.21%)	0.0200 (1st)
WLS	0.7656 (6.20%)	0.8312 (3.30%)	0.7191 (19.20%)	0.6343 (18.34%)	0.6815 (1.17%)	0.8063 (1.34%)	0.1423 (− 14.20%)	0.0270 (− 43.33%)	0.2019 (4th)
CSR	0.7795 (4.31%)	0.8497 (1.05%)	0.8152 (5.15%)	0.6655 (12.79%)	0.6897 (− 0.03%)	0.8072 (1.23%)	0.1284 (− 4.91%)	0.0334 (− 54.19%)	17.5814 (10th)
LRD	0.7575 (7.34%)	0.8058 (6.55%)	0.7319 (17.12%)	0.6559 (14.44%)	0.6737 (2.35%)	0.8067 (1.29%)	0.1577 (− 22.57%)	0.0232 (− 34.05%)	33.5066 (11th)
TLayers	0.6375 (27.55%)	0.6997 (22.71%)	0.5648 (51.77%)	0.5696 (31.78%)	0.5999 (14.94%)	0.8067 (1.29%)	0.2595 (− 52.95%)	0.0330 (− 53.64%)	0.6608 (6th)
CSMCA	0.8029 (1.27%)	0.8489 (1.14%)	0.7903 (8.47%)	0.6878 (9.13%)	0.6643 (3.79%)	0.8063 (1.34%)	0.1537 (− 20.56%)	0.0193 (− 20.73%)	57.9753 (12th)
LATLRR	0.7196 (12.99%)	0.7041 (21.94%)	0.7971 (7.54%)	0.7398 (1.46%)	0.6175 (11.66%)	0.8065 (1.31%)	0.1639 (− 25.50%)	0.0158 (− 3.16%)	7.9590 (8th)
DTNP	0.7905 (2.86%)	0.8489 (1.14%)	0.8291 (3.39%)	0.6961 (7.83%)	0.6671 (3.36%)	0.8064 (1.33%)	0.1239 (− 1.45%)	0.0322 (− 52.48%)	14.0796 (9th)
VANet	0.8131 (1st)	0.8586 (1st)	0.8572 (1st)	0.7506 (1st)	0.6895 (2nd)	0.8171 (1st)	0.1221 (1st)	0.0153 (1st)	0.1649 (3rd)

#### Cavernous angioma

**Fig. 12:**
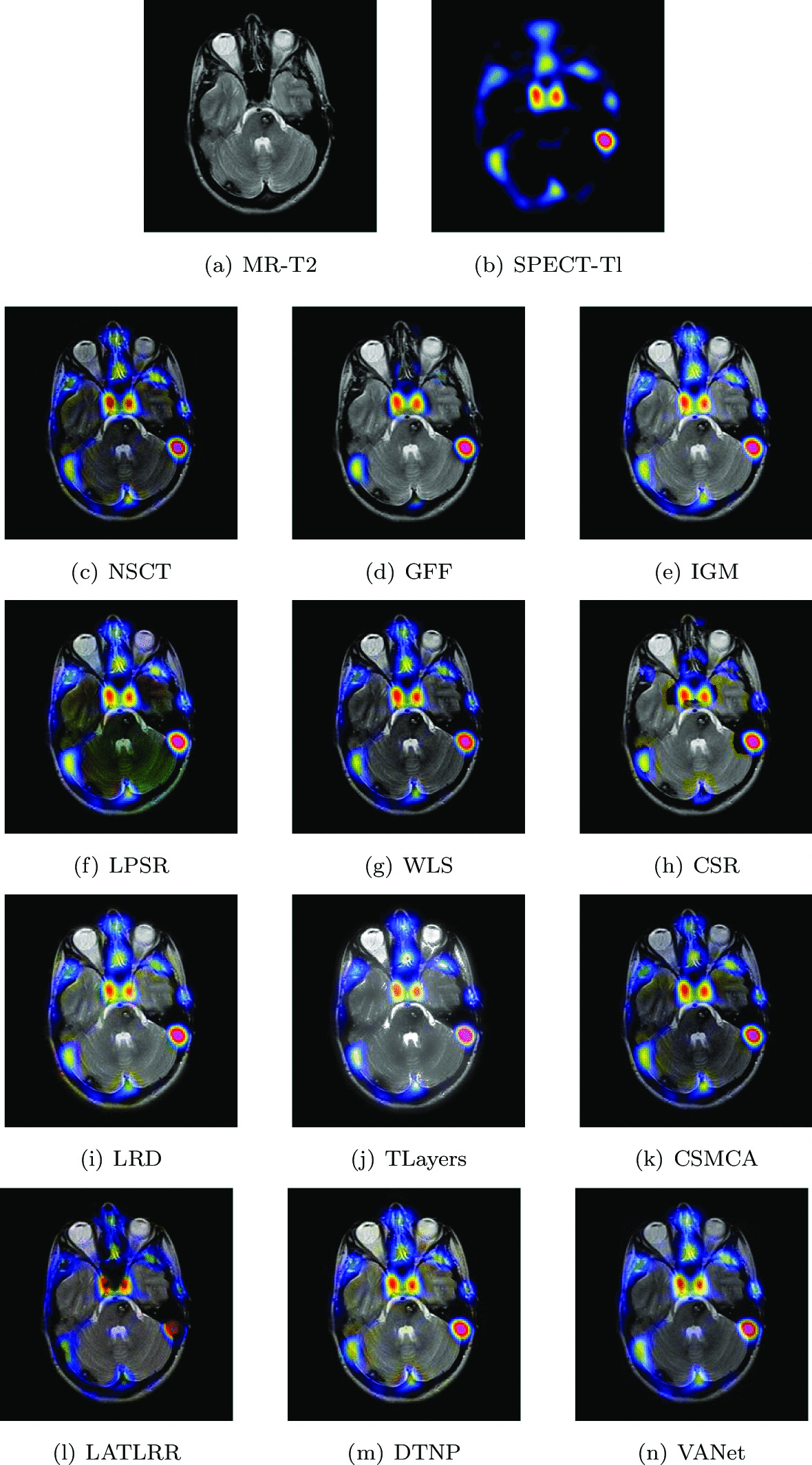
The first set of fused MRI-SPECT images from 9 methods on cavernous angioma

**Fig. 13:**
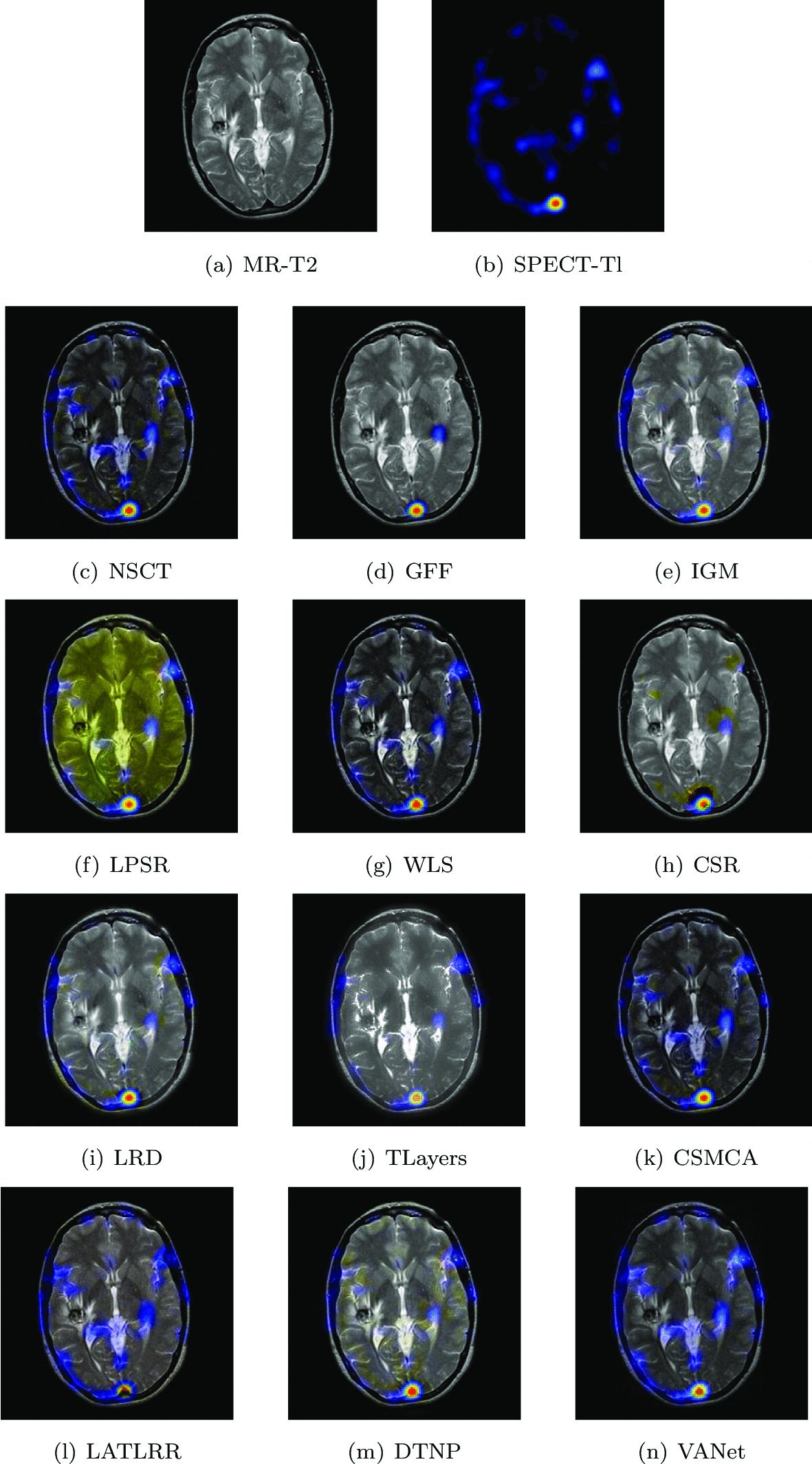
The second set of fused MRI-SPECT images from 9 methods on cavernous angioma

The experimental data is from a 26-year-old woman with a ten-year history of headaches. Recently, she received radiosurgery due to progressive weakness of the right arm and leg. Her MR images show obvious hemangiomas. Her SPECT image is marked with technetium. Among them are blood clots and scarred brains, surrounded by crystalline old blood products. The lesion can not fill the marked red blood cells, indicating that they are not open to circulating blood. In order to assist the doctor in completing the diagnosis and treatment of her disease better, her two sets of registered images were chosen to be fused. Figures 12 and 13 show the fusion results of two sets of images under different algorithms, respectively. The fused images obtained based on NSCT, WLS and CSMCA algorithms lack the low-frequency energy of the SPECT image, resulting in its dim brightness. The fused image obtained by LPSR algorithm is seriously distorted. The brightness of the fused image obtained by IGM, LRD and DTNP algorithms is too high, which affects the description of the texture information. The fused images obtained based on GFF, CSR and LATLRR algorithms describe the blood circulation process poorly. The fused image obtained by TLayers algorithm is relatively blurry and can not describe the nuclide information. The fused image obtained by VANet model is superior to other algorithms in terms of brightness, contrast and description of nuclide information.

Tables 5 and 6 show the objective representation of all algorithms on the above two sets of images, respectively. With the exception of VIF and Qe, VANet achieves optimal solutions on all other metrics. Although the images obtained by IGM algorithm and DTNP algorithm are optimally solved in terms of visual fidelity and Qe metrics, respectively. Their poor performance in the fusion results has seriously affected the doctor’s observation of texture details. In summary, the fused images obtained by VANet model can help doctors complete to observe and diagnose glioma diseases better.

**Table 5:** The objective evaluation scores about group 5 fused images

Methods	Metrics								
	*Q*_*w*_	*Q*_*e*_	SSIM	VIF	FMI	NCIE	LABF	NABF	Time
NSCT	0.6587 (14.86%)	0.7006 (9.08%)	0.5287 (23.45%)	0.4139 (18.50%)	0.5287 (32.59%)	0.8034 (1.47%)	0.1494 (− 9.71%)	0.0215 (− 59.07%)	0.4724 (5th)
GFF	0.6701 (12.91%)	0.7315 (4.47%)	0.6062 (7.67%)	0.3778 (29.80%)	0.6284 (11.55%)	0.8087 (0.80%)	0.1402 (− 3.78%)	0.0144 (− 38.89%)	0.0578 (2nd)
IGM	0.7298 (3.67%)	0.7205 (6.06%)	0.6385 (2.22%)	0.5036 (− 2.60%)	0.6965 (0.65%)	0.8064 (1.09%)	0.1410 (− 4.33%)	0.0185 (− 52.43%)	2.0005 (7th)
LPSR	0.7047 (7.36%)	0.7125 (7.26%)	0.6219 (4.95%)	0.4595 (6.75%)	0.6657 (5.30%)	0.8033 (1.48%)	0.1618 (− 16.63%)	0.0231 (− 61.90%)	0.0247 (1st)
WLS	0.7045 (7.40%)	0.7083 (7.89%)	0.6056 (7.78%)	0.4619 (6.19%)	0.6773 (3.50%)	0.8042 (1.37%)	0.1405 (− 3.99%)	0.0209 (− 57.89%)	0.1582 (3rd)
CSR	0.7030 (7.62%)	0.7414 (3.08%)	0.6436 (1.41%)	0.3838 (27.80%)	0.6903 (1.55%)	0.8061 (1.13%)	0.1354 (− 0.37%)	0.0148 (− 40.54%)	17.4229 (10th)
LRD	0.7205 (5.01%)	0.6979 (9.50%)	0.5983 (9.09%)	0.4746 (3.35%)	0.6525 (7.43%)	0.8049 (1.28%)	0.1572 (− 14.19%)	0.0185 (− 52.43%)	33.8562 (11th)
TLayers	0.5773 (31.06%)	0.5562 (37.40%)	0.4715 (38.43%)	0.4370 (12.24%)	0.5808 (20.70%)	0.8046 (1.32%)	0.2029 (− 33.51%)	0.0206 (− 57.28%)	0.5873 (6th)
CSMCA	0.6918 (9.37%)	0.7160 (6.73%)	0.5484 (19.02%)	0.4331 (13.25%)	0.6678 (4.97%)	0.8038 (1.42%)	0.1578 (− 14.51%)	0.0146 (− 39.73%)	51.0000 (12th)
LATLRR	0.6370 (18.78%)	0.6201 (23.24%)	0.5052 (29.20%)	0.4660 (5.26%)	0.6163 (13.74%)	0.8044 (1.34%)	0.2482 (− 45.65%)	0.0133 (− 33.83%)	8.1679 (8th)
DTNP	0.7465 (1.35%)	0.7563 (1.04%)	0.6407 (1.87%)	0.4816 (1.85%)	0.6605 (6.13%)	0.8046 (1.32%)	0.1536 (− 12.17%)	0.0221 (− 60.18%)	13.8429 (9th)
VANet	0.7566 (1st)	0.7642 (1st)	0.6527 (1st)	0.4905 (2nd)	0.7010 (1st)	0.8152 (1st)	0.1349 (1st)	0.0088	0.1549 (4th)
								(1st)

**Table 6:** The objective evaluation scores about group 6 fused images

Methods	Metrics								
	*Q*_*w*_	*Q*_*e*_	SSIM	VIF	FMI	NCIE	LABF	NABF	Time
NSCT	0.7484 (16.02%)	0.8219 (9.32%)	0.6544 (22.65%)	0.6402 (27.87%)	0.5931 (23.22%)	0.8042 (1.80%)	0.0823 (− 39.00%)	0.0125 (− 58.40%)	0.4863 (5th)
GFF	0.8299 (4.63%)	0.8976 (0.10%)	0.7024 (14.27%)	0.7604 (7.65%)	0.7281 (0.37%)	0.8156 (0.38%)	0.0610 (− 17.70%)	0.0106 (− 50.94%)	0.0581 (2nd)
IGM	0.8450 (2.76%)	0.8872 (1.27%)	0.7128 (12.60%)	0.8046 (1.74%)	0.6976 (4.76%)	0.8107 (0.99%)	0.0639 (− 21.44%)	0.0111 (− 53.15%)	1.9547 (7th)
LPSR	0.8537 (1.71%)	0.8762 (2.55%)	0.7949 (0.97%)	0.7977 (2.62%)	0.6733 (8.54%)	0.8049 (1.71%)	0.0554 (− 9.39%)	0.0123 (− 57.72%)	0.0265 (1st)
WLS	0.7715 (12.55%)	0.8268 (8.67%)	0.7131 (12.55%)	0.7568 (8.17%)	0.6401 (14.17%)	0.8049 (1.71%)	0.0707 (− 29.00%)	0.0108 (− 51.85%)	0.1880 (4th)
CSR	0.8479 (2.41%)	0.8976 (0.10%)	0.7185 (11.70%)	0.7564 (8.22%)	0.6674 (9.50%)	0.8083 (1.29%)	0.0636 (− 21.07%)	0.0134 (− 61.19%)	16.7486 (10th)
LRD	0.8175 (6.21%)	0.8342 (7.71%)	0.6873 (16.78%)	0.7523 (8.81%)	0.6032 (21.15%)	0.8058 (1.60%)	0.0851 (− 40.41%)	0.0055 (− 5.45%)	33.5259 (11th)
TLayers	0.6448 (34.66%)	0.6804 (32.05%)	0.5475 (46.59%)	0.7318 (11.86%)	0.5420 (34.83%)	0.8053 (1.66%)	0.1068 (− 53.00%)	0.0101 (− 48.51%)	0.5913 (6th)
CSMCA	0.7560 (14.85%)	0.8245 (8.98%)	0.6417 (25.07%)	0.6641 (23.26%)	0.6468 (12.99%)	0.8044 (1.78%)	0.0826 (− 39.23%)	0.0088 (− 40.90%)	52.2731 (12th)
LATLRR	0.7278 (19.30%)	0.7980 (12.59%)	0.6670 (20.33%)	0.6053 (35.24%)	0.6416 (13.90%)	0.8048 (1.73%)	0.0952 (− 47.27%)	0.0095 (− 45.26%)	8.0273 (8th)
DTNP	0.8610 (0.85%)	0.8989 (− 0.04%)	0.7486 (7.21%)	0.7926 (3.28%)	0.6768 (7.98%)	0.8077 (1.36%)	0.0506 (− 0.79%)	0.0133 (− 60.90%)	14.3408 (9th)
VANet	0.8683 (1st)	0.8985 (2nd)	0.8026 (1st)	0.8186 (1st)	0.7308 (1st)	0.8187 (1st)	0.0502 (1st)	0.0052 (1st)	0.1653 (3rd)

#### Metastatic bronchogenic carcinoma

**Fig. 14:**
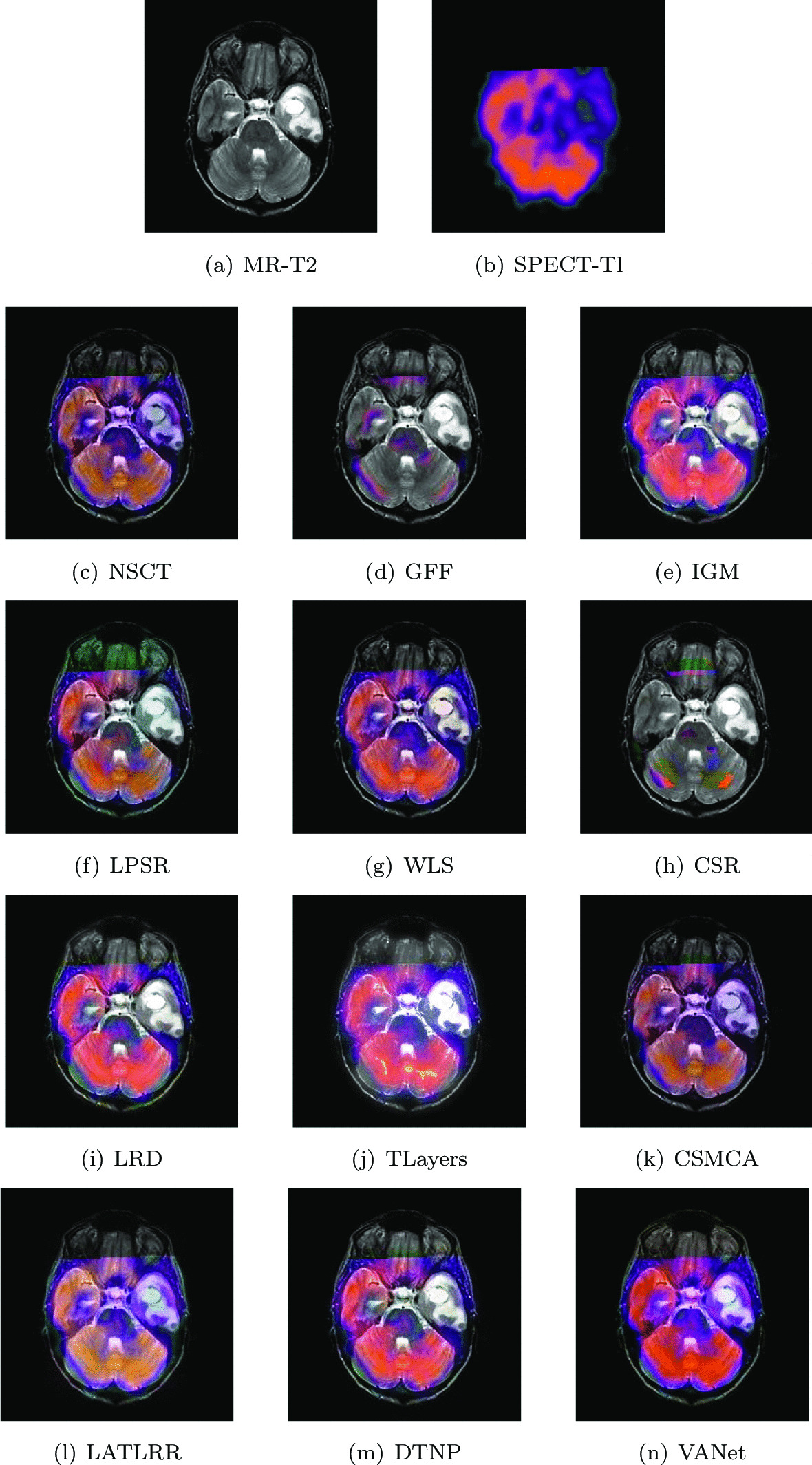
The first set of fused MRI-SPECT images from 9 methods on metastatic bronchogenic carcinoma

**Fig. 15:**
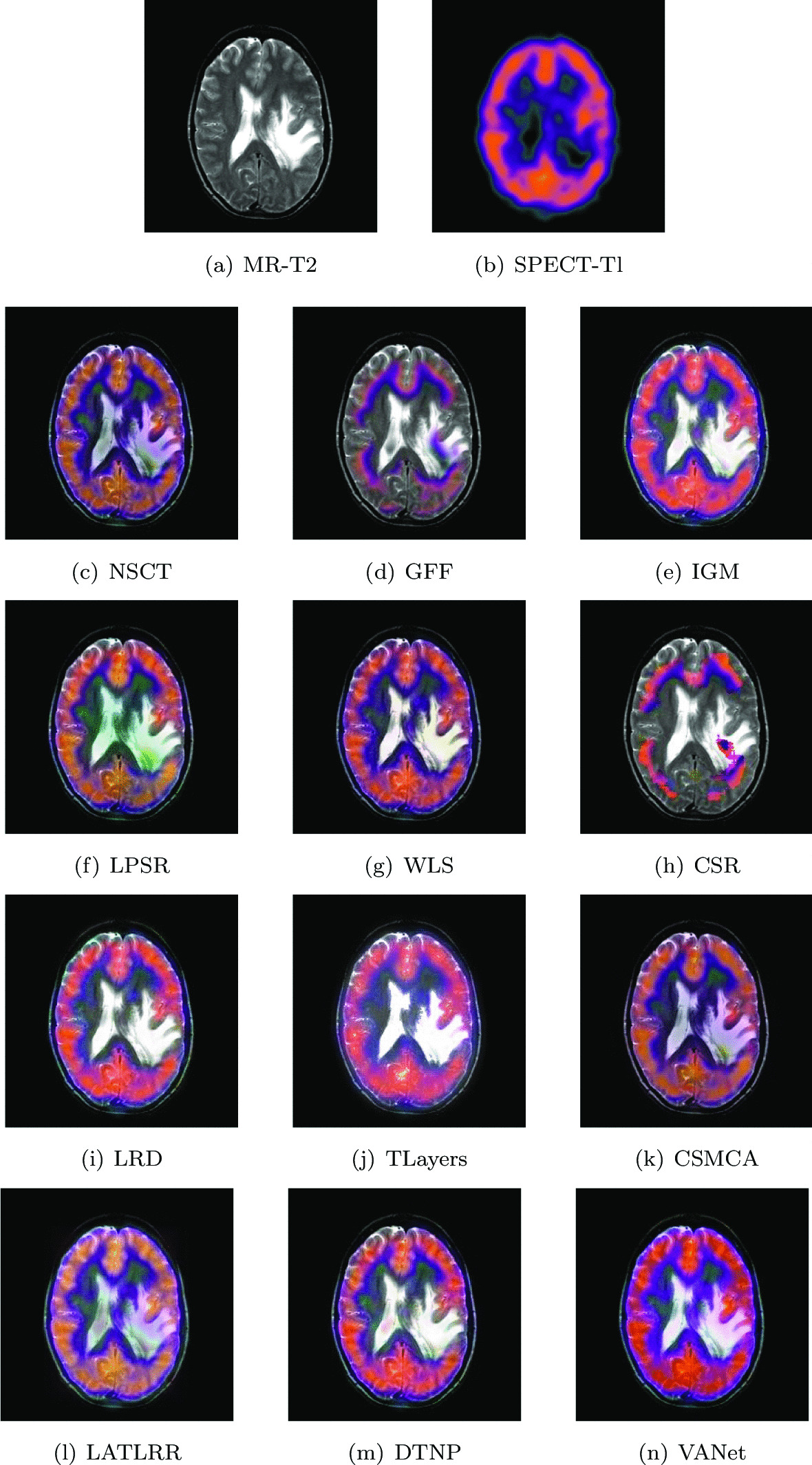
The second set of fused MRI-SPECT images from 9 methods on metastatic bronchogenic carcinoma

The experimental data comes from a 42-year-old woman who has been smoking for a long time and the sudden increase in headaches caused her to go to the hospital for a check-up. After examination, a large number of lumps appeared in her brain. The MR image demonstrates the tumor as an area of high signal intensity on proton density (PD) and T2-weighted (T2) images in a large left temporal region. Perfusion SPECT image shows very low blood flow to the lesion. In order to further combine tissue structure information and blood flow conditions to accelerate the diagnostic process, two sets of registered medical images are selected for fusion. Figures 14 and 15 show the fusion results of two sets of images under different algorithms, respectively. The fused image obtained by TLayers algorithm is blurred in texture detail. The fused images obtained based on NSCT and CSMCA algorithms have a dim brightness and lose the low-frequency energy in the SPECT image. The fused images obtained by LPSR and LATLRR algorithms show color distortion. The fused images obtained based on GFF and CSR algorithms lose the ability to describe the blood flow levels of tissues. The brightness of the fused images obtained by IGM, WLS, LRD and DTNP algorithms is too large, which seriously affects the expression of image color information. The fused image obtained by VANet model has a appropriate contrast and can help doctors judge the adhesion relationship between brain tissue and metastatic cancers.

Tables 7 and 8 show the objective representations of all fusion results of these two sets of images, respectively. The VANet model has a significant performance improvement over other algorithms, except the LPSR algorithm. Although the LPSR algorithm and the VANet model perform equally well on all metrics, the images obtained by LPSR algorithm describe color information very poorly. In summary, the VANet model is more suitable for processing image fusion of bronchial cancer metastatic disease, which can provide great help to doctors.

**Table 7:** The objective evaluation scores about group 7 fused images

Methods	Metrics								
	*Q*_*w*_	*Q*_*e*_	SSIM	VIF	FMI	NCIE	LABF	NABF	Time
NSCT	0.7509 (20.14%)	0.8181 (5.55%)	0.6777 (11.04%)	0.5685 (14.35%)	0.6642 (10.07%)	0.8047 (1.55%)	0.1030 (− 15.92%)	0.0502 (− 59.96%)	0.4689 (5th)
GFF	0.7665 (17.69%)	0.8449 (2.20%)	0.6652 (13.12%)	0.4967 (30.88%)	0.7013 (4.25%)	0.8099 (0.90%)	0.1012 (− 14.43%)	0.0234 (− 14.10%)	0.0493 (2nd)
IGM	0.7006 (28.76%)	0.7303 (18.24%)	0.6630 (13.50%)	0.5555 (17.03%)	0.6748 (8.34%)	0.8058 (1.41%)	0.1656 (− 47.71%)	0.0205 (− 1.95%)	2.0595 (7th)
LPSR	0.8925 (1.08%)	0.8493 (1.67%)	0.7439 (1.16%)	0.5919 (9.83%)	0.6994 (4.53%)	0.8055 (1.45%)	0.0930 (− 6.88%)	0.0272 (− 26.10%)	0.0291 (1st)
WLS	0.7348 (22.77%)	0.7947 (8.66%)	0.6723 (11.93%)	0.5562 (16.88%)	0.7000 (4.44%)	0.8052 (1.49%)	0.1122 (− 22.82%)	0.0268 (− 25.00%)	0.4363 (4th)
CSR	0.7924 (13.84%)	0.8525 (1.29%)	0.6861 (9.68%)	0.5013 (29.68%)	0.7216 (1.32%)	0.8087 (1.05%)	0.0998 (− 13.23%)	0.0264 (− 23.86%)	15.2319 (10th)
LRD	0.7346 (22.80%)	0.7702 (12.11%)	0.6780 (10.99%)	0.5789 (12.30%)	0.6733 (8.58%)	0.8059 (1.40%)	0.1319 (− 34.34%)	0.0255 (− 21.18%)	33.7116 (11th)
TLayers	0.6140 (44.92%)	0.6327 (36.48%)	0.5154 (46.00%)	0.5052 (28.68%)	0.5979 (22.28%)	0.8056 (1.44%)	0.2092 (− 58.60%)	0.0233 (− 13.73%)	0.5990 (6th)
CSMCA	0.7661 (17.75%)	0.8310 (3.91%)	0.6494 (15.88%)	0.5457 (19.13%)	0.6941 (5.33%)	0.8051 (1.50%)	0.1125 (− 23.02%)	0.0291 (− 30.93%)	52.2022 (12th)
LATLRR	0.6683 (34.98%)	0.6577 (31.29%)	0.6807 (10.55%)	0.6404 (1.51%)	0.6402 (14.20%)	0.8053 (1.48%)	0.1557 (− 44.38%)	0.0221 (− 9.05%)	8.0074 (8th)
DTNP	0.7707 (17.05%)	0.8253 (4.63%)	0.7343 (2.48%)	0.6077 (6.98%)	0.6898 (5.99%)	0.8058 (1.41%)	0.0876 (− 1.14%)	0.0281 (− 28.47%)	14.7256 (9th)
VANet	0.9021 (1st)	0.8635 (1st)	0.7525 (1st)	0.6501 (1st)	0.7311 (1st)	0.8172 (1st)	0.0866 (1st)	0.0201 (1st)	0.1682 (3rd)

**Table 8:** The objective evaluation scores about group 8 fused images

Methods	Metrics								
	*Q*_*w*_	*Q*_*e*_	SSIM	VIF	FMI	NCIE	LABF	NABF	Time
NSCT	0.7198 (8.38%)	0.7889 (4.61%)	0.6766 (14.32%)	0.5852 (18.46%)	0.6610 (10.53%)	0.8058 (1.35%)	0.1347 (− 7.13%)	0.0309 (− 29.13%)	0.4892 (5th)
GFF	0.6909 (12.91%)	0.8037 (2.69%)	0.5700 (35.70%)	0.5854 (18.41%)	0.6503 (12.35%)	0.8083 (1.04%)	0.1428 (− 12.39%)	0.0311 (− 29.58%)	0.0519 (2nd)
IGM	0.7345 (6.21%)	0.7079 (16.58%)	0.7045 (9.79%)	0.6300 (10.03%)	0.6548 (11.58%)	0.8067 (1.24%)	0.2042 (− 38.74%)	0.0248 (− 11.69%)	2.0109 (7th)
LPSR	0.7703 (1.27%)	0.8153 (1.23%)	0.7619 (− 1.10%)	0.6425 (7.89%)	0.6979 (4.69%)	0.8063 (1.29%)	0.1287 (− 2.80%)	0.0277 (− 20.94%)	0.0194 (1st)
WLS	0.7548 (3.35%)	0.7954 (3.76%)	0.7350 (2.52%)	0.6220 (11.45%)	0.6976 (4.73%)	0.8066 (1.25%)	0.1341 (− 6.71%)	0.0348 (− 37.07%)	0.1492 (3rd)
CSR	0.7190 (8.50%)	0.8114 (1.71%)	0.6851 (9.98%)	0.5134 (35.02%)	0.7199 (1.49%)	0.8085 (1.01%)	0.1314 (− 4.79%)	0.0427 (− 48.71%)	14.1277 (10th)
LRD	0.7659 (1.85%)	0.7926 (4.13%)	0.7408 (1.71%)	0.6434 (7.74%)	0.6906 (5.79%)	0.8071 (1.19%)	0.1476 (− 15.24%)	0.0283 (− 22.61%)	33.3678 (11th)
TLayers	0.6722 (16.05%)	0.6572 (25.58%)	0.6010 (25.37%)	0.5653 (22.63%)	0.6069 (20.38%)	0.8068 (1.23%)	0.2768 (− 50.80%)	0.0307 (− 28.66%)	0.5858 (6th)
CSMCA	0.7364 (5.93%)	0.8006 (3.09%)	0.6697 (12.51%)	0.6004 (15.46%)	0.6644 (9.96%)	0.8062 (1.30%)	0.1509 (− 17.10%)	0.0226 (− 3.09%)	36.0721 (12th)
LATLRR	0.6630 (17.66%)	0.6412 (28.71%)	0.6851 (9.98%)	0.6830 (1.49%)	0.6242 (17.03%)	0.8062 (1.30%)	0.2045 (− 38.83%)	0.0233 (− 6.01%)	7.8907 (8th)
DTNP	0.7593 (2.74%)	0.8044 (2.60%)	0.7531 (0.05%)	0.6357 (9.05%)	0.6706 (8.95%)	0.8065 (1.26%)	0.1272 (− 1.65%)	0.0353 (− 37.96%)	14.0789 (9th)
VANet	0.7801 (1st)	0.8253 (1st)	0.7535 (2nd)	0.6932 (1st)	0.7306 (1st)	0.8167 (1st)	0.1251 (1st)	0.0219 (1st)	0.1579 (4th)

#### Mild Alzheimer’s disease

**Fig. 16:**
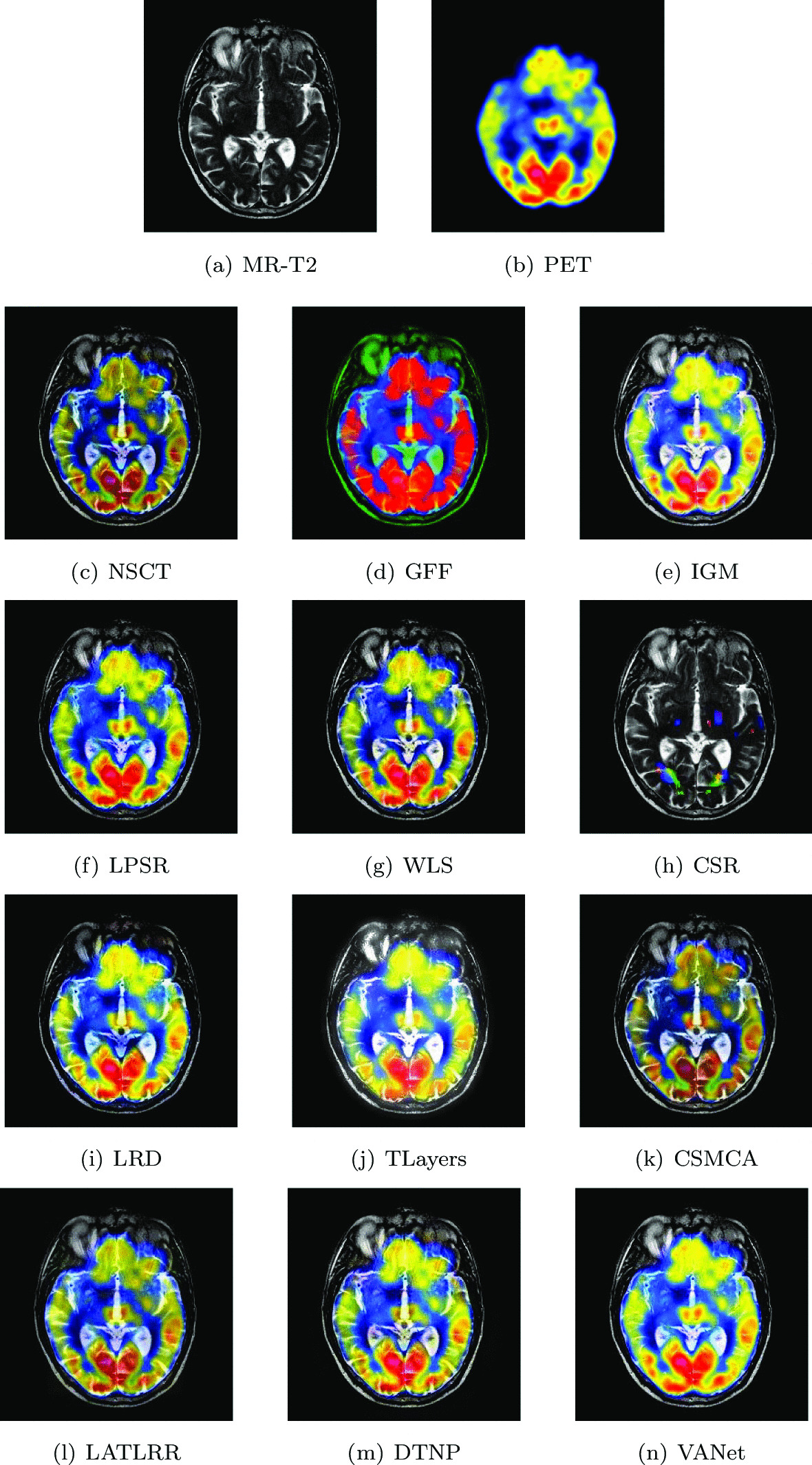
The first set of fused MRI-PET images from 12 methods on mild Alzheimer’s disease

**Fig. 17:**
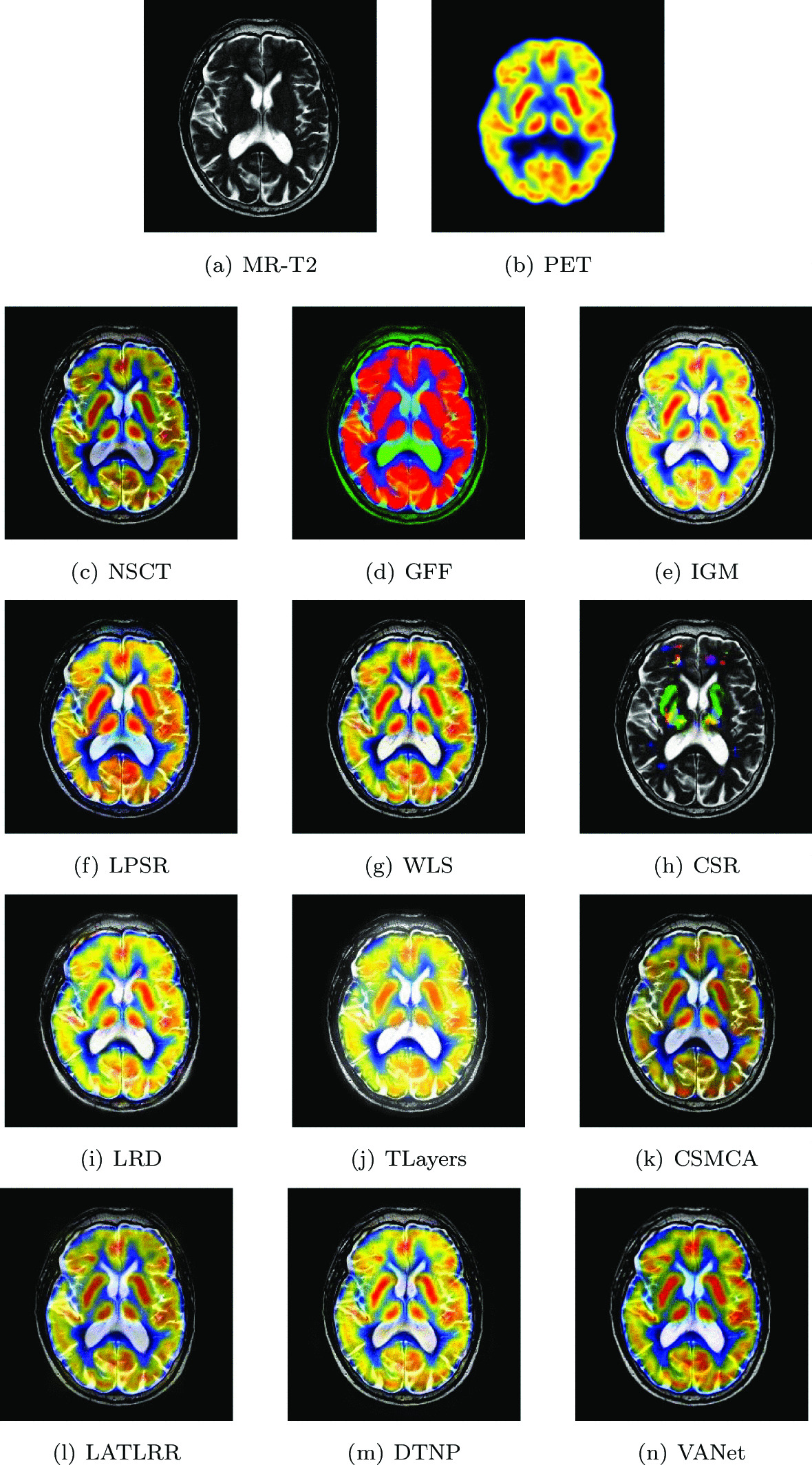
The second set of fused MRI-PET images from 12 methods on mild Alzheimer’s disease

The experimental images are taken from a 70-year-old man with memory difficulties.MR images showed globally widened hemispheric sulci, which is more prominent in parietal lobes. In his PET images, regional cerebral metabolism is markedly abnormal with hypometabolism in anterior temporal and posterior parietal regions. To further observe the metabolic status of the tumor location, his two sets of images are removed for fusion. Figures 16 and 17 show all the fusion results of the two sets of images, respectively. The brightness of the fused images obtained based on NSCT and CSMCA algorithms is too dark and the energy information of the PET image is lost. The image obtained by CSR algorithm loses almost all metabolic information. The fused image obtained by GFF algorithm shows serious color distortion. In the fused images obtained by IGM, DTNP and WLS algorithms, the brightness of them is too high, resulting in loss of information. The fused images obtained by LRD, LPSR and LATLRR algorithms have low contrast in the upper right corner and the outline is not obvious. The fused image obtained by TLayers algorithm has a severe blurry texture. The fused image obtained by VANet model can contain rich texture information and complete metabolic information.

Tables 9 and 10 show the objective performance of the two sets of images in different fusion algorithms. Compared with other algorithms, the VANet model achieves suboptimal values on the FMI metric and performs best on the remaining metrics. Combined with the fusion result, the medical images fused by VANet model can provide great help to doctors in the process of treating mild Alzheimer’s disease.

**Table 9:** The objective evaluation scores about group 8 fused images

Methods	Metrics								
	*Q*_*w*_	*Q*_*e*_	SSIM	VIF	FMI	NCIE	LABF	NABF	Time
NSCT	0.6619 (5.03%)	0.7205 (4.34%)	0.5230 (18.66%)	0.3252 (23.37%)	0.5823 (17.19%)	0.8041 (1.77%)	0.1627 (− 5.16%)	0.0203 (− 29.56%)	0.4960 (5th)
GFF	0.5010 (38.76%)	0.6234 (20.60%)	0.4489 (38.25%)	0.2924 (37.21%)	0.5768 (18.31%)	0.8040 (1.78%)	0.1681 (− 8.20%)	0.0141 (1.42%)	(2nd) 0.0576
IGM	0.6933 (0.27%)	0.6888 (9.14%)	0.6100 (1.74%)	0.3427 (17.07%)	0.6593 (3.50%)	0.8058 (1.55%)	0.1946 (− 20.70%)	0.0169 (− 15.38%)	2.1689 (7th)
LPSR	0.6702 (3.73%)	0.6314 (19.07%)	0.5468 (13.50%)	0.3356 (19.55%)	0.6295 (8.40%)	0.8044 (1.73%)	0.2195 (− 29.70%)	0.0199 (− 28.14%)	0.0287 (1st)
WLS	0.6945 (0.10%)	0.7113 (5.69%)	0.5794 (7.11%)	0.3299 (21.61%)	0.6332 (7.77%)	0.8046 (1.70%)	0.1689 (− 8.64%)	0.0216 (− 33.80%)	0.3690 (4th)
CSR	0.5427 (28.10%)	0.7421 (1.30%)	0.5168 (20.09%)	0.2623 (52.95%)	0.6826 (− 0.03%)	0.8078 (1.30%)	0.1745 (− 11.58%)	0.0219 (− 34.70%)	(10th) 16.0802
LRD	0.6594 (5.43%)	0.6651 (13.04%)	0.5523 (12.37%)	0.2968 (35.18%)	0.6162 (10.74%)	0.8043 (1.74%)	0.1920 (− 19.64%)	0.0328 (− 56.40%)	34.4963 (11th)
TLayers	0.5511 (26.15%)	0.6077 (23.71%)	0.5020 (23.63%)	0.3219 (24.63%)	0.5406 (26.23%)	0.8042 (1.75%)	0.2835 (− 45.57%)	0.0152 (− 5.92%)	0.6978 (6th)
CSMCA	0.6476 (7.35%)	0.7360 (2.15%)	0.5095 (21.81%)	0.3072 (30.60%)	0.6455 (5.72%)	0.8045 (1.71%)	0.1617 (− 4.58%)	0.0161 (− 11.18%)	48.1738 (12th)
LATLRR	0.61550 (12.95%)	0.6219 (20.89%)	0.4556 (36.22%)	0.3935 (1.96%)	0.5997 (17.79%)	0.8047 (1.69%)	0.1983 (− 22.19%)	0.0173 (− 17.34%)	7.9532 (8th)
DTNP	0.6888 (0.93%)	0.7171 (4.84%)	0.5754 (7.86%)	0.3496 (14.76%)	0.6080 (12.24%)	0.8048 (1.68%)	0.1549 (− 0.39%)	0.0223 (− 35.87%)	14.8684 (9th)
VANet	0.6952 (1st)	0.7518 (1st)	0.6206 (1st)	0.4012 (1st)	0.6824 (2nd)	0.8183 (1st)	0.1543 (1st)	0.0143 (2nd)	0.1702 (3rd)

**Table 10:** The objective evaluation scores about group 9 fused images

Methods	Metrics								
	*Q*_*w*_	*Q*_*e*_	SSIM	VIF	FMI	NCIE	LABF	NABF	Time
NSCT	0.6196 (6.71%)	0.6521 (8.28%)	0.4803 (17.34%)	0.3041 (25.35%)	0.6051 (24.03%)	0.8041 (1.26%)	0.1813 (− 4.69%)	0.0184 (− 33.70%)	0.5007 (5th)
GFF	0.5237 (26.26%)	0.5326 (32.57%)	0.5235 (7.66%)	0.2902 (31.36%)	0.7507 (− 0.03%)	0.8041 (1.26%)	0.2208 (− 21.74%)	0.0143 (− 14.69%)	0.0588 (2nd)
IGM	0.6499 (1.74%)	0.6212 (13.67%)	0.5451 (3.39%)	0.3047 (25.11%)	0.6594 (13.82%)	0.8056 (1.07%)	0.2274 (− 24.01%)	0.0136 (− 10.29%)	2.1402 (7th)
LPSR	0.6362 (3.93%)	0.6200 (13.89%)	0.4923 (14.48%)	0.2959 (28.83%)	0.6360 (18.00%)	0.8043 (1.23%)	0.2014 (− 14.20%)	0.0184 (− 33.70%)	0.0299 (1st)
WLS	0.6581 (0.47%)	0.6496 (8.70%)	0.5311 (6.12%)	0.2988 (27.58%)	0.6417 (16.95%)	0.8047 (1.18%)	0.1918 (− 9.91%)	0.0188 (− 35.11%)	0.3217 (4th)
CSR	0.4737 (39.58%)	0.6853 (3.04%)	0.5151 (9.42%)	0.2782 (37.02%)	0.6853 (9.51%)	0.8073 (0.85%)	0.1935 (− 10.70%)	0.0241 (− 49.38%)	15.9263 (10th)
LRD	0.6306 (4.85%)	0.5689 (24.12%)	0.4794 (17.56%)	0.2857 (33.43%)	0.5982 (25.46%)	0.8046 (1.19%)	0.2465 (− 29.90%)	0.0165 (− 26.06%)	34.3784 (11th)
TLayers	0.4955 (33.44%)	0.5391 (30.98%)	0.4427 (27.31%)	0.3121 (22.14%)	0.5526 (35.81%)	0.8047 (1.18%)	0.3045 (− 43.25%)	0.0128 (− 4.69%)	0.7025 (6th)
CSMCA	0.5997 (10.26%)	0.6884 (2.57%)	0.4814 (17.08%)	0.2915 (30.77%)	0.6579 (14.08%)	0.8046 (1.19%)	0.1726 (1.16%)	0.0141 (− 13.48%)	44.3954 (12th)
LATLRR	0.5703 (15.94%)	0.5673 (24.47%)	0.4253 (32.52%)	0.3641 (4.70%)	0.5921 (26.75%)	0.8048 (1.17%)	0.2246 (− 23.06%)	0.0169 (− 27.81%)	8.0570 (8th)
DTNP	0.6508 (1.60%)	0.6474 (9.07%)	0.5099 (10.53%)	0.3212 (18.68%)	0.5796 (29.49%)	0.8047 (1.18%)	0.1798 (− 3.89%)	0.0192 (− 36.46%)	15.0684 (9th)
VANet	0.6612 (1st)	0.7061 (1st)	0.5636 (1st)	0.3812 (1st)	0.7505 (2nd)	0.8142 (1st)	0.1728 (2nd)	0.0122 (1st)	0.1759 (3rd)

#### Ablation study

**Fig. 18:**
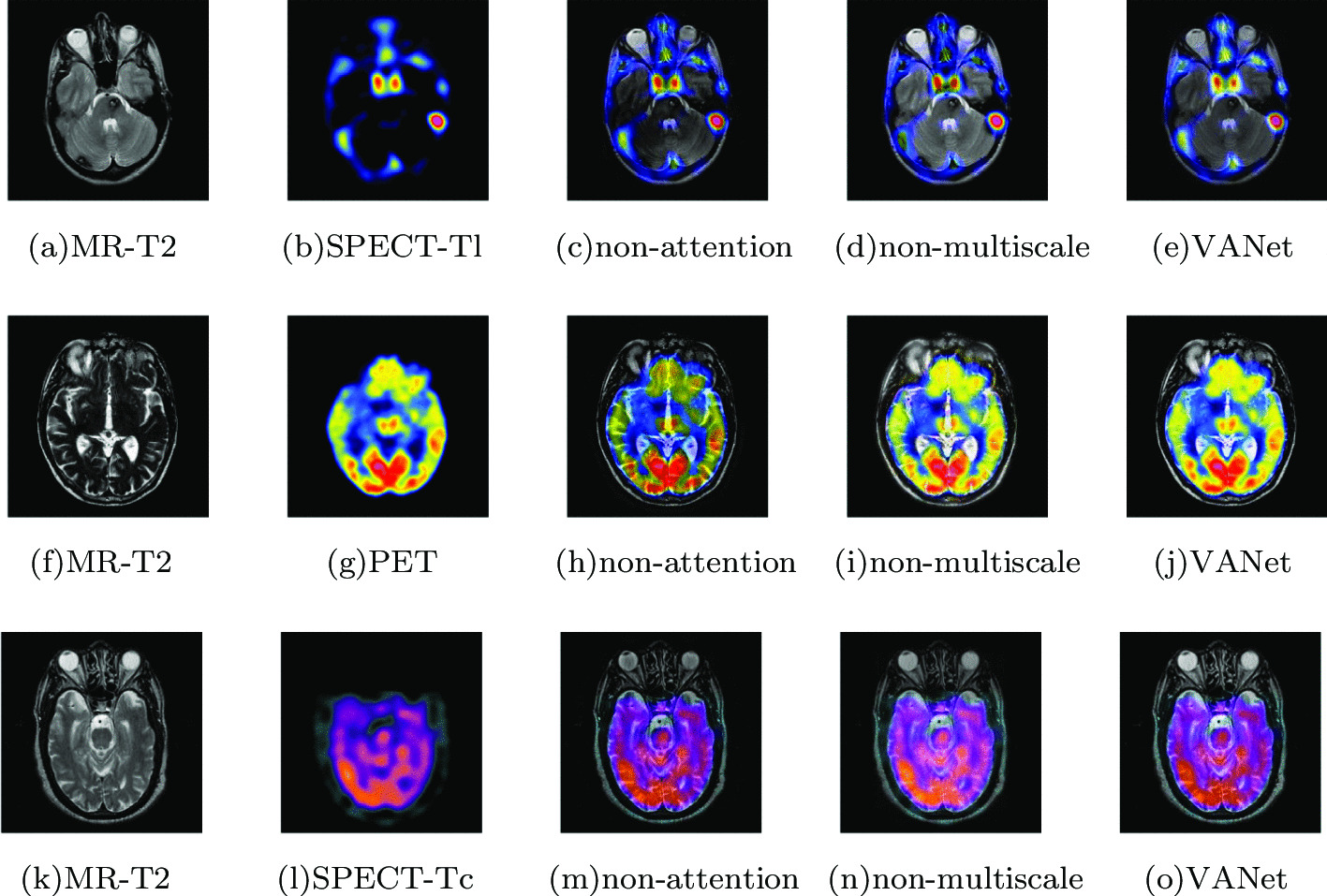
Influence of attention mechanism branch and residual multi-scale detail processing branch in VANet model on fusion results respectively

The core of the VANet model is the attention-multiscale fusion network. Among them, the attention mechanism branch is to fuse the global context of medical images; the residual multi-scale detail processing branch is to fuse the local context of medical images. In order to verify the influence of the two branches on the fusion results, the section chooses to ignore one of the branches and use the other branch for fusion. The experimental data are 60 groups of registered MRI and their corresponding nuclear medicine images, from which we randomly select the fusion results of three groups of images and show them in Fig. 18.

First, in order to verify the influence of global context on the fused images, the attention mechanism branch is ignored. The fusion results are shown in Fig. 8c, h and m. When the global context fusion is blocked, the fused image suffers from severe color distortion, resulting in a large deviation in the description of tissue metabolic information. Then, the residual multi-scale detail branch is ignored to verify the effect of local context on the fused images. The fusion results are shown in Fig. 8d, i and n. It can be clearly found that some detailed texture information is blurred, which affects the doctor’s observation of key tissue information. Table 1 shows the statistical results of objective metrics of the VANet model ablation experiment and the optimal value is selected in bold.

**Table 11:** The objective evaluation scores about group 10 fused images

Methods	Metrics								
		*Q*_*w*_	*Q*_*e*_	SSIM	VIF	FMI	NCIE	LABF	NABF
1	Non-attention	0.7893	0.7953	0.6770	0.5371	0.6796	0.8063	0.1403	0.0127
	Non-multiscale	0.8072	0.8154	0.6965	0.5146	0.7066	0.8086	0.1287	0.0198
	VANet	0.8231	0.8297	0.7048	0.5642	0.7126	0.8125	0.0947	0.0063
2	Non-attention	0.6582	0.7126	0.5219	0.2977	0.6234	0.8121	0.1876	0.0223
	Non-multiscale	0.6473	0.6972	0.5312	0.3109	0.6324	0.8098	0.1768	0.0267
	VANet	0.6675	0.7304	0.5429	0.3343	0.6588	0.8168	0.1584	0.0126
3	Non-attention	0.8124	0.8452	0.8494	0.5954	0.6201	0.8112	0.1945	0.0218
	Non-multiscale	0.7993	0.8344	0.8561	0.6137	0.6315	0.8095	0.1232	0.0253
	VANet	0.8329	0.8603	0.8752	0.6318	0.6682	0.8163	0.1014	0.0189

In Table 11, it can be seen that the performance of the VANet model with the attention branch removed is significantly weaker on most metrics, especially in SSIM, FMI, and LABF. It shows that the global context plays an important role in the medical image fusion. The VANet model with the residual multiscale detail processing branch removed has the worst performance on the metric of NABF, which indicates that the local context affects the description of detail information in the fused image. Without this branch, the fused image would have more noisy information. In contrast, the complete VANet model considers the representation of image global information and local information, which improves the quality of fused images.

#### Time complexity analysis

The image obtained by the VAnet model has been subjectively analyzed and objectively evaluated before. This section will evaluate the VANet model and other algorithms from the perspective of time complexity. The time cost of each algorithm on each set of experimental images has been shown in Tables 1 to 10. From all the tables, it can be found that the LPSR algorithm takes the shortest time and the CSMCA algorithm takes the longest time. The time consumption of the LRD mehod is second only to the CSMCA algorithm. The time consumption of the CSR method and the DTMP algorithm also exceeded 10 seconds. The VAnet model takes some time to train. After the model is trained, the time it takes to fuse images is comparable to that of the WLS algorithm. However, the fusion effect of the VANet is much better than the WLS and the LPSR algorithms.

#### Statistical test

**Fig. 19:**
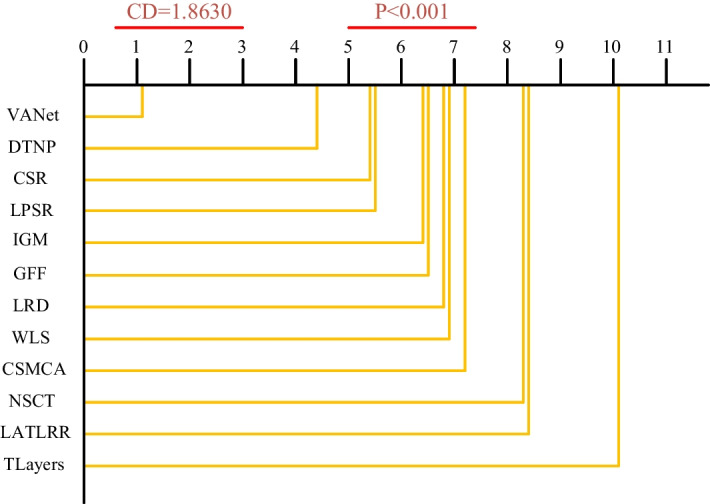
The time complexity of different types of medical images

When comparing algorithms, it is often necessary to perform statistical tests on experimental results. Friedman test is a type of nonparametric test used to measure the performance of multiple algorithms on different datasets. However, Friedman test can only detect whether there are differences between the performance of multiple algorithms. Once there is a difference, a post-hoc test is needed to find out which algorithms have statistical differences in their performance. Nermenyi test is a commonly used method for subsequent testing. It uses Tukey’s distribution to complete the critical difference (CD) calculation. The level difference of any two methods is larger than the value of CD, which proves that there is a significant difference between the two methods. In Fig. 19, the values of the objective evaluation indicators in Tables 1, 2, 3, 4, 5, 6, 7, 8, 9, 10 are used to calculate the level of each fusion algorithm. Combining the above two test results, we can find that the VANet model has obvious performance advantages compared with other fusion algorithms. In the evaluation of the selected objective indicators, the VAnet model has certain statistical significance.

## Conclusions

In this study, we propose a novel fusion model for medical image fusion. Aiming at the challenges faced by medical image fusion, first, the model uses the five blocks of VGG-16 to build an encoder to obtain feature maps containing image context information. Second, the model constructs an AM fusion network with the attention mechanism as the core. The network builds blocks around the channel attention mechanism to enhance salient features and weaken redundant features. In order to get more texture details, the network uses different convolution kernels to construct detail information patches to obtain multi-scale features of the image. Finally, all the acquired features are reconstructed by the decoder. The experimental results on the Harvard Medical School brain medical image dataset show that the fused images obtained by the VAnet model are superior to the current more advanced fusion algorithms in terms of structural information and metabolic condition expression. Since the VAnet model can avoid the problem of image fusion sequences, it can be further extended to the field of three medical images fusion.

## Data Availability

The datasets used and/or analyzed during the current study are available from https://www.med.harvard.edu/AANLIB/home.html Experimental images in Fig. 1 are downloaded from https://www.med.harvard.edu/AANLIB/cases/caseNN1/mr1-dg1/015.html Experimental images in Fig. 8 are downloaded from https://www.med.harvard.edu/AANLIB/cases/case15/mr1-tc1/012.html Experimental images in Fig. 9 are downloaded from https://www.med.harvard.edu/AANLIB/cases/case15/mr1-tc1/015.html Experimental images in Fig. 10 are downloaded from https://www.med.harvard.edu/AANLIB/cases/case21/mr1-tc1/009.html Experimental images in Fig. 11 are downloaded from https://www.med.harvard.edu/AANLIB/cases/case21/mr1-tc1/016.html Experimental images in Fig. 12 are downloaded from https://www.med.harvard.edu/AANLIB/cases/case12/mr2-tc2/007.html Experimental images in Fig. 13 are downloaded from https://www.med.harvard.edu/AANLIB/cases/case12/mr2-tc2/023.html Experimental images in Fig. 14 are downloaded from https://www.med.harvard.edu/AANLIB/cases/case28/mr1-tc1/006.html Experimental images in Fig. 15 are downloaded from https://www.med.harvard.edu/AANLIB/cases/case28/mr1-tc1/013.html.

## Abbreviations

VGG-16: Visual Geometry Group network with 16 layers
MR: Magnetic resonance
PET: Positron emission tomography
AE: Autoencoders
CNN: Convolutional neural network
GAN: Generative Adversarial Network
PD: Proton density
T2: T2-weighted
SSIM: Structural similarity
AG: Average gradient
VIF: Visual information fidelity
FMI: :Future mutual information
MSE: Mean square error
LPSR: Laplacian pyramid sparse representation
LRD: Laplacian re-decomposition
SPECT: Single-Photon Emission Computed Tomography
MSDNet: Multi-Scale Dense Convolutional Networks
TAcGAN: Tissue-aware conditional generative adversarial network
TV: Total variation
WLS: Weighted least square optimization
CSR: Convolutional sparse representation
TLayers: Three-layer medical image fusion
CSMCA: Medical image fusion via convolutional sparsity based morphological component analysis
LATLRR: Latent low-rank representation
DTNP: Dynamic threshold neural p systems medical image fusion
NCIE: Nonlinear correlation information entropy.

## References

[1] Fu J, Li W, Du J, Xu L (2021). Dsagan: a generative adversarial network based on dual-stream attention mechanism for anatomical and functional image fusion. Inf Sci.

[2] Ganasala P, Prasad AD (2020). Medical image fusion based on laws of texture energy measures in stationary wavelet transform domain. Int J Imaging Syst Technol.

[3] Singh S, Gupta D, Anand R, Kumar V (2015). Nonsubsampled shearlet based ct and mr medical image fusion using biologically inspired spiking neural network. Biomed Signal Process Control.

[4] Shahdoosti HR, Mehrabi A (2018). Multimodal image fusion using sparse representation classification in tetrolet domain. Digit Signal Process.

[5] Shahdoosti HR, Mehrabi A (2018). Mri and pet image fusion using structure tensor and dual ripplet-ii transform. Multimed Tools Appl.

[6] Li S, Kang X, Fang L, Hu J, Yin H. Pixel-level image fusion: a survey of the state of the art. Inf Fusion. 2017;33:100–112.

[7] Wang Q, Li S, Qin H, Hao A (2015). Robust multi-modal medical image fusion via anisotropic heat diffusion guided low-rank structural analysis. Inf Fusion.

[8] Liu S, Liu S, Cai W, Che H, Pujol S, Kikinis R, Feng D, Fulham MJ (2014). Multimodal neuroimaging feature learning for multiclass diagnosis of alzheimer’s disease. IEEE Trans Biomed Eng.

[9] Shi B, Chen Y, Zhang P, Smith CD, Liu J, Initiative ADN (2017). Nonlinear feature transformation and deep fusion for Alzheimer’s disease staging analysis. Pattern Recognit.

[10] Singh V, Verma NK, Ul Islam Z, Cui Y. Feature learning using stacked autoencoder for shared and multimodal fusion of medical images. In: Verma, G.A.K. Nishchal K. (ed.) Computational Intelligence: Theories, Applications and Future Directions-Volume I.

[11] Goodfellow I, Pouget-Abadie J, Mirza M, Xu B, Warde-Farley D, Ozair S, Courville A, Bengio Y. Generative adversarial nets. Adv Neural Inf Process Syst. 2014.

[12] Tang W, Liu Y, Cheng J, Li C, Chen X (2021). Green fluorescent protein and phase contrast image fusion via detail preserving cross network. IEEE Trans Comput Imaging.

[13] Liu Y, Chen X, Cheng J, Peng H. A medical image fusion method based on convolutional neural networks. In: 2017 20th International Conference on Information Fusion (Fusion). 2017:1–7.

[14] Hermessi H, Mourali O, Zagrouba E (2018). Convolutional neural network-based multimodal image fusion via similarity learning in the shearlet domain. Neural Comput Appl.

[15] Xia K-j, Yin H-s, Wang J-q. A novel improved deep convolutional neural network model for medical image fusion. Cluster Comput. 2019;22(1);1515–1527.

[16] Song X, Wu X-J, Li H. Msdnet for medical image fusion. In: International Conference on Image and Graphics. 2019:278–288.

[17] Kang J, Lu W, Zhang W (2020). Fusion of brain pet and mri images using tissue-aware conditional generative adversarial network with joint loss. IEEE Access.

[18] Zhang Y, Liu Y, Sun P, Yan H, Zhao X, Zhang L (2020). Ifcnn: a general image fusion framework based on convolutional neural network. Inf Fusion.

[19] Li S, Kang X, Hu J (2013). Image fusion with guided filtering. IEEE Trans Image Process.

[20] Li T, Wang Y (2011). Biological image fusion using a nsct based variable-weight method. Inf Fusion.

[21] Zhang X, Li X, Feng Y, Zhao H, Liu Z (2015). Image fusion with internal generative mechanism. Expert Syst Appl.

[22] Wang Z, Cui Z, Zhu Y (2020). Multi-modal medical image fusion by laplacian pyramid and adaptive sparse representation. Comput Biol Med.

[23] Ma J, Zhou Z, Wang B, Zong H (2017). Infrared and visible image fusion based on visual saliency map and weighted least square optimization. Infrared Phys Technol.

[24] Liu Y, Chen X, Ward RK, Wang ZJ (2016). Image fusion with convolutional sparse representation. IEEE Signal Process Lett.

[25] Li X, Guo X, Han P, Wang X, Li H, Luo T (2020). Laplacian redecomposition for multimodal medical image fusion. IEEE Trans Instrum Meas.

[26] Du J, Li W, Tan H (2020). Three-layer medical image fusion with tensor-based features. Inf Sci.

[27] Liu Y, Chen X, Ward RK, Wang ZJ (2019). Medical image fusion via convolutional sparsity based morphological component analysis. IEEE Signal Process Lett.

[28] Li H, Wu X-J. Infrared and visible image fusion using latent low-rank representation. arXiv preprint arXiv:1804.08992. 2018.

[29] Li B, Peng H, Wang J (2021). A novel fusion method based on dynamic threshold neural p systems and nonsubsampled contourlet transform for multi-modality medical images. Signal Process.

[30] Piella G, Heijmans H. A new quality metric for image fusion. In: Proceedings 2003 International Conference on Image Processing (Cat. No. 03CH37429), 2003;3:173.

[31] Zhou W. Image quality assessment: from error measurement to structural similarity. IEEE Trans Image Process. 2004. 10.1109/tip.2003.819861 15376593

[32] Han Y, Cai Y, Cao Y, Xu X (2013). A new image fusion performance metric based on visual information fidelity. Inf Fusion.

[33] Haghighat M, Razian MA. Fast-fmi: non-reference image fusion metric. In: 2014 IEEE 8th International Conference on Application of Information and Communication Technologies (AICT). 2014:1–3.

[34] Petrovic V, Xydeas C. Objective image fusion performance characterisation. In: Tenth IEEE International Conference on Computer Vision (ICCV’05). 2005;Volume 1, vol. 2:1866–1871.

[35] Shreyamsha Kumar B (2013). Multifocus and multispectral image fusion based on pixel significance using discrete cosine harmonic wavelet transform. Signal Image Video Process.

[36] Wang Q, Shen Y, Jin J (2008). Performance evaluation of image fusion techniques. Image Fusion Algorithms Appl.

